# Myelin sheath lengths in the central nervous system scale to axon diameter via oligodendroglial Piezo1

**DOI:** 10.1371/journal.pbio.3003992

**Published:** 2026-09-21

**Authors:** Amanda R. Young, Ashley Galfano, Jacob Reyngoudt, Ryan W. Lewis, Martha Cash, Beckam Polis, Myah Zalusky, Avipsha Datta, Marie E. Bechler

**Affiliations:** 1 Department of Cell and Developmental Biology, SUNY Upstate Medical University, Syracuse, New York, United States of America; 2 Department of Neuroscience and Physiology, SUNY Upstate Medical University, Syracuse, New York, United States of America; TU Munich: Technische Universitat Munchen, GERMANY

## Abstract

Myelin sheath lengths vary by an order of magnitude in the central nervous system (CNS) and tune the timing of neuronal signaling. Thus, variation in myelin sheath length has been proposed to coordinate the timing of neuronal signaling to ultimately impact behavior. The mechanisms to establish myelin sheath length are unknown. For decades, reports have documented that in vivo myelin sheath size scales with the diameter of the ensheathed axon. We previously demonstrated diameter is sufficient to instruct myelin sheath lengths formed by rat oligodendrocytes using a synthetic axon culture system. The mechanisms of oligodendrocyte diameter-sensing and its translation into sheath elongation are still unknown. Here, we demonstrate that diameter-sensing and sheath length is locally regulated: each individual myelin sheath responds to the underlying fiber diameter. We uncover a novel mechanism for scaling myelin sheath length to fiber diameter, through mechanosensitive ion channel Piezo1. In mice in vivo, Piezo1 impacts the elongation of myelin sheaths on large diameter axons, recapitulating our in vitro results. Yet, surprisingly, there is no impact on myelin thickness with conditional Piezo1 loss. We propose Piezo1 provides a mechanism to establish hard-wired myelin sheath patterns, where oligodendrocytes transduce axon diameter into generating myelin segments with vastly different lengths.

## Abstract 2

How oligodendrocytes match their myelin production to the scale of the axon they ensheathe is poorly understood. This study shows that oligodendrocytes require Piezo1 to appropriately scale sheath lengths to axon diameter, supporting the temporal coordination of neuronal signaling.

## Introduction

Myelin sheaths support the health and energy efficiency of axons [1] as well as impact the speed of action potential propagation along axons. Signalling speed in myelinated axons greatly depends on the physical properties of the myelin sheaths, especially sheath length (i.e., internodal distance) [2,3]. Strikingly, the CNS displays an order of magnitude variation in myelin sheath lengths, from 10s to 100s of micrometers in length [4–6]. Together, this has spurred the hypothesis that variation in myelin sheath lengths is critical to CNS function by precisely adjusting neuronal signaling speeds to facilitate signal synchronization within neuronal circuits [7,8]. Indeed, tuning myelin sheath size to coordinate signal timing may be a fundamental mechanism to enable behavior [9–11] and can be perturbed in an array of neurodevelopmental [12,13] and neurodegenerative conditions [14]. What governs how CNS myelin sheath lengths are initially established is still unclear.

For decades, in vivo studies have demonstrated a nearly linear positive correlation between myelin sheath lengths and axon diameters for the majority of myelinated axons [15–17]. How oligodendrocytes scale myelin sheath lengths with axon diameter is unknown. Studies have pointed to a major role for diameter sensing in both onset of myelin sheath formation as well as myelin sheath lengths. Reductionist approaches with synthetic axons (fibers that mimic the size and shape of axons) demonstrated that a threshold fiber diameter is sufficient to trigger myelination [18]. Complementary in vivo studies also showed that increased axon diameter can stimulate unmyelinated axons to become myelinated [19]. Consistent with diameter being sufficient to stimulate myelination, in vivo data has demonstrated myelination can occur independently of active neuronal signals [20–23]. Direct evidence that diameter also instructs sheath length was demonstrated with isolated oligodendrocytes on synthetic axons, where myelin sheath lengths scaled with the diameter of synthetic axons [24]. Taken all together, these results led to the proposed model that oligodendrocytes sense and use axon diameter as a cue to initiate oligodendrocytes’ intrinsic ability to form myelin sheaths and establish CNS myelin sheath lengths, setting a baseline of ‘hard-wired’ myelin sheath formation that can be modified further by other extrinsic signals such as neuronal activity [25]. In such a model, the molecular pathways sensing and responding to axon diameter are anticipated to be some of the main drivers of neuronal-activity-independent myelin sheath growth. In this work, we sought to identify how diameter (axon or microfiber) is sensed as a physical cue and translated into control of myelin sheath lengths.

Mechanotransduction, the ability to sense and convert physical cues into cellular responses, is an increasingly appreciated but understudied property of many cell types, including oligodendrocyte lineage cells. Physical cues such as extracellular stiffness affect oligodendrocyte lineage cell differentiation and can have opposing effects on myelination. Increased substrate stiffness promotes changes to transcription factor localization, can activate transcriptional and/or epigenetic changes, as well as alter oligodendrocyte lineage differentiation [26–30]. Multiple mechanotransduction pathways including LINC complex, YAP/TAZ, integrin, and mechanosensitive ion channel signalling have all been implicated in oligodendrocyte maturation [26,28,31–35]. Interestingly, mechanotransduction during myelin formation appears to be regulated opposingly to oligodendrocyte differentiation: pliable substrates promote myelin membrane wrapping of synthetic axons while wrapping is hindered by stiff substrates [30]. Further, recent evidence suggests that mechanosensitive channels such as TMEM63 regulate the formation of myelin on small (< 2 micrometer) diameter axons [35]. Emerging evidence of mechanotransduction in oligodendrocytes highlights the important, open question of how differentiated oligodendrocytes integrate physical cues to regulate myelin sheath formation and properties. The mechanotransduction pathways that drive the formation of spirally wrapped myelin membranes are still poorly understood.

Here we investigate how oligodendrocytes sense axon diameters and translate this physical signal into myelin sheath lengths. We establish that diameter sensing is transduced at the individual myelin sheath level and identify a mechanosensitive protein responsible for myelin sheath elongation on large diameter axons.

## Results

### Myelin sheath lengths scale locally with axon diameter

To determine the mechanisms that oligodendrocytes use to scale myelin sheath growth (lengths) with the physical cue of diameter, we have taken advantage of oligodendrocyte cultures with electrospun microfibers [36]. These microfibers mimic the size and shape of CNS axons and are sufficient to promote isolated rodent oligodendrocytes to form compact, multilamellar myelin sheaths that mirror myelin sheath lengths observed in vivo [24]. An important strength of this model is the ability to tightly control culture conditions and fiber diameter, thereby eliminating any influences from the CNS biochemical milieu. Oligodendrocytes cultured on uniform microfibers of specific diameter ranges (0.5–1 micrometer, 1–2 micrometers, or 2–4 micrometers) scale myelin sheath lengths to the diameter of the fibers [24], mimicking observed in vivo correlations of myelin sheath length with axon diameter [15–17]. This neuron-free system is ideal for elucidating how axon diameter is translated into myelin sheath lengths, because it enables testing the contribution of diameter as the sole variable during the 3-D process of myelin sheath formation.

**Fig 1:**
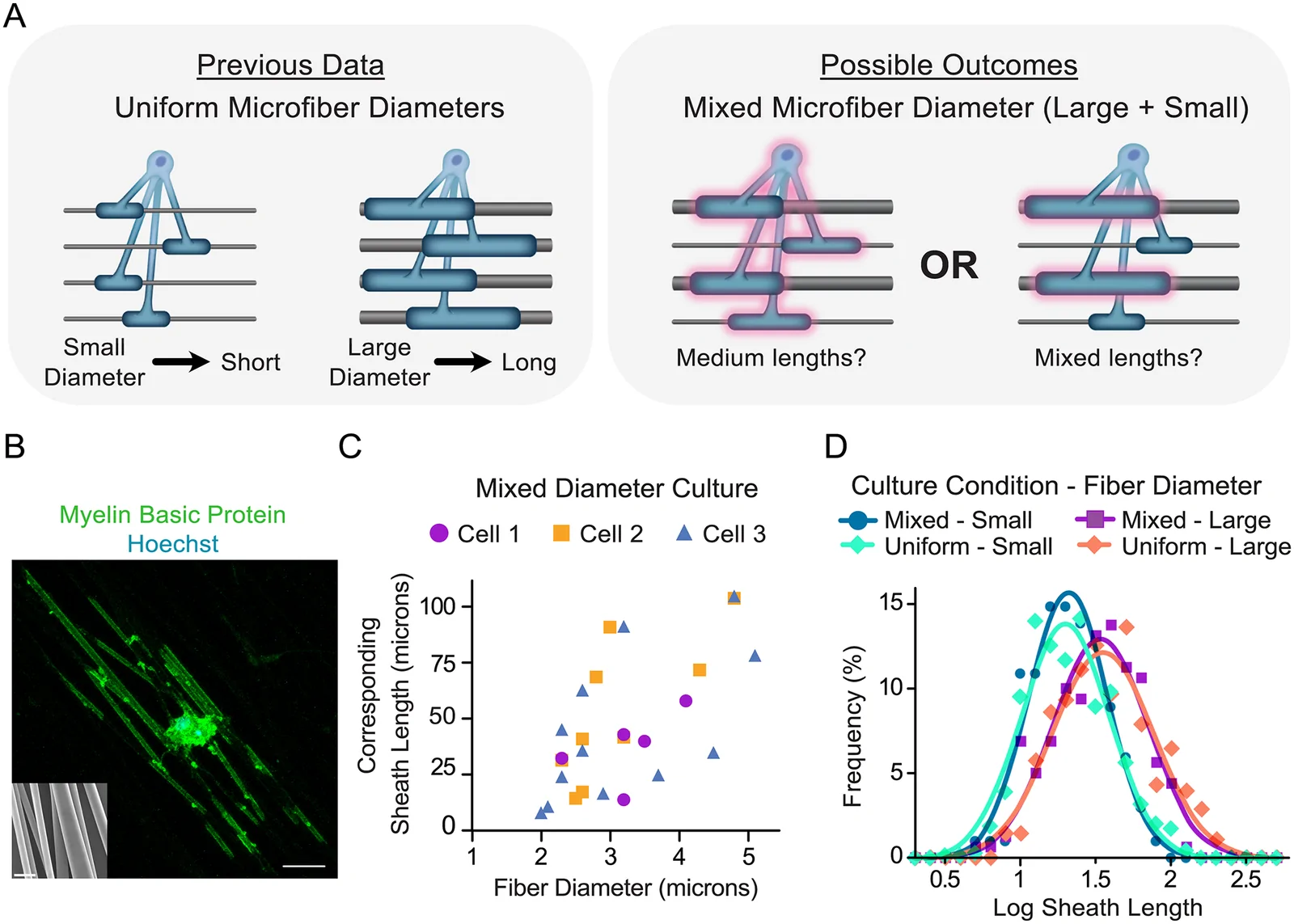
Each nascent myelin sheath locally senses and responds to fiber diameter. **A)** Experiment and predictions. Oligodendrocytes cultured on uniform fiber diameters generate short myelin sheaths on small diameter fibers and long myelin sheaths on large diameter fibers. When oligodendrocytes contact both small and large diameter fibers simultaneously, they will adjust sheath length at the whole cell level (medium lengths) or locally within each nascent myelin sheath (same sheath lengths as they form on uniform fiber diameters). **B)** Confocal maximum projection image of an oligodendrocyte on mixed diameter fibers, forming myelin sheaths on both large and small diameter fibers simultaneously. Scale = 20 µm. SEM inset shows mixed diameter microfibers, scale = 5 µm. **C)** Myelin sheath lengths plotted with the corresponding fiber diameter for three example oligodendrocytes. **D)** Frequency distribution of the log of myelin sheath lengths formed, binned by fiber diameter: small (1–2.5 micrometer) diameter fibers from mixed diameters (solid line, dark blue) or uniform fiber diameter cultures (dashed line, light blue-green), and myelin sheaths formed on large diameter fibers (2.6–6 micrometers) on mixed diameters on (solid line, purple) or uniform (dashed line orange). >63 individual oligodendrocytes analyzed, from *n* = 3 independent experiments each with pooled rat litters (>127 sheaths on small diameters, >309 sheaths on large diameters). The distribution of myelin sheath lengths is statistically different between fiber diameters but unchanged between uniform or mixed diameters (*t* test, *p* = 0.42 for small diame*t*er uniform vs. mixed, *p* = 0.21 for large diameter uniform vs. mixed, *p* < 0.01 comparing large vs. small). The data underlying this figure can be found under the doi: https://doi.org/10.6084/m9.figshare.33116207 in the following FigShare collection: https://figshare.com/s/6e5a23b8d1765ff06f5a.

First, we addressed whether oligodendrocytes respond to fiber diameter on a whole-cell level or if every individual nascent myelin sheath locally regulates length in response to diameter. Mechanotransduction in oligodendrocyte lineage cells has previously been shown to involve YAP/TAZ or LINC complex signalling that mediates transcriptional or epigenetic changes [26,30,31]. In contrast to this, individual oligodendrocytes can generate myelin sheaths that vary in size, suggesting local regulation [4,37,38]. To distinguish between global (e.g., transcriptional) or local responses, we modified the design of our microfiber system to generate mixed diameter microfiber scaffolds (Fig 1A and 1B). Mixed diameter microfibers enabled assessment of myelin sheaths formed by single oligodendrocytes simultaneously in contact with both small and large diameter fibers. We predicted that if signalling led to whole-cell responses, the myelin sheaths lengths would no longer scale with diameter in mixed diameter fiber cultures. However, if sheath length is locally regulated, we predicted that sheath lengths would scale with diameter, equivalent to myelin sheaths in uniform-diameter microfiber cultures (Fig 1A).

Myelin sheath length in response to underlying fiber diameter was assessed from primary rat oligodendrocytes cultured on mixed diameter microfibers for 14 days, when maximum sheath lengths are formed [24]. Each myelin sheath length and the corresponding fiber diameter was measured from single oligodendrocytes simultaneously in contact with both large and small diameter fibers, using confocal micrographs (Figs 1B and S1). We observed that myelin sheath lengths increased with the underlying fiber diameter (Fig 1C), supporting that each nascent myelin sheath length locally scales with the diameter of the fiber. To confirm whether myelin sheath lengths are locally controlled in each nascent myelin sheath on a larger scale, we binned a total of 127 myelin sheath lengths formed on small diameter (0.5–2.5 micrometers) and large diameter fibers (2.6–6 micrometers, S1A and S1B Fig). We compared the binned myelin sheath length distribution from mixed diameter cultures against the distribution of myelin sheath lengths in uniform-diameter microfibers. The distribution of myelin sheath lengths between mixed diameter and uniform diameter microfiber cultures were statistically equivalent (Fig 1D, data shown per replicate in S1C Fig), consistent with a model where myelin sheath lengths are controlled at the level of individual sheaths.

### Piezo1 enables local myelin sheath length scaling to diameter

**Fig 2:**
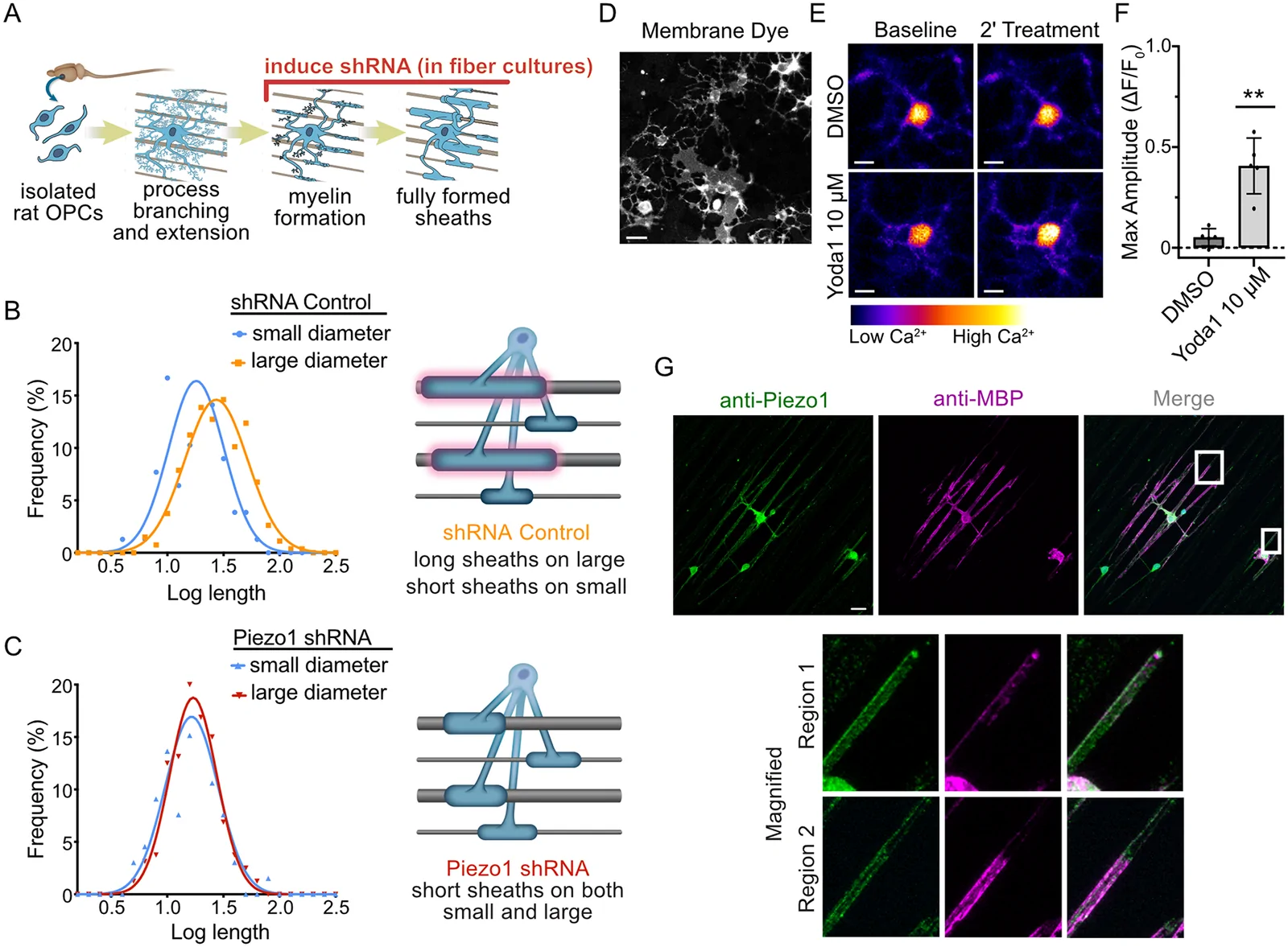
Piezo1 is present in nascent myelin sheaths and is important for myelin sheath elongation on large diameter fibers. **A)** Experimental schematic: shRNA-mediated knockdown of membrane tension-sensing candidate Piezo1 or control shRNA expression was induced after OL differentiation and process extension in mixed diameter fiber cultures. **B)** Log normal curves and schematic representation of myelin sheath length distributions on small or large diameter fibers from mixed diameter cultures with control shRNA. Sheath lengths on large diameter fibers are longer compared to those on small diameter fibers. *p* = 0.038, Welch’s corrected *t* test of mean log sheath length on small vs. large diameter fibers. *n* = 4 independent fiber cultures with oligodendrocytes from different pooled rat litters. **C)** Log normal curves and schematic of myelin sheath length distributions of sheaths formed on small or large diameter fibers from mixed diameter cultures with Piezo1 shRNA. Sheath length scaling with diameter is lost with Piezo1 shRNA-mediated knockdown. *p* = 0.64, Welch’s corrected *t* test of mean log sheath length on small vs. large diameter fibers. *n* = 4 independent fiber cultures with oligodendrocytes from different pooled rat litters. **D)** Maximum projected image of membrane dye-labelled differentiated rat OLs used for Yoda1 calcium imaging. **E)** Fluo-8-loaded OLs treated with DMSO (top) or 10 µM Yoda1 (bottom) at baseline (left) or after 2 min of corresponding treatment (right). Scale bar = 10 µm. **F)** Amplitude (maximum change in fluorescence intensity) of intracellular calcium upon treatment. *n* = 5 independent cell isolations, *t* test with Welch’s correction, *p* = 0.003. Bars = mean ± standard deviation. **G)** Maximum projected immunofluorescence image of differentiated oligodendrocytes after 3 days on 2-micrometer fibers. Magnified regions show localization within and extending beyond forming sheaths. Scale = 20 µm. The data underlying this figure can be found under the DOIs: https://doi.org/10.6084/m9.figshare.33116285, https://doi.org/10.6084/m9.figshare.33166640 in the following FigShare collection: https://figshare.com/s/6e5a23b8d1765ff06f5a.

We next set out to address how the diameter is integrated by oligodendrocytes to generate different myelin sheath lengths. When nascent myelin sheaths wrap around axons, myelin membrane curvatures would vary substantially between small diameter (0.5–2.5 micrometers) and large diameter (2.5–5 micrometers) axons. Many mechanosensory proteins generate local signaling in response to membrane bending and membrane tension forces; therefore, we cross-referenced mechanosensory proteins in the literature with oligodendrocyte expression profiles [28,39,40] to generate a list of candidates. We examined the contribution of these candidate proteins to diameter-responsive myelin sheath elongation by utilizing doxycycline-inducible knockdown in primary cortical rat oligodendrocytes cultured on mixed-diameter microfibers. shRNA expression was induced after initial differentiation (based on myelin basic protein (MBP) expression), when initial ensheathment has begun: 4–5 days in culture on microfibers (Figs 2A and S2A–S2H). Myelin sheath lengths and the underlying fiber diameters were measured from oligodendrocytes in contact with both large and small diameter microfibers. As expected, control shRNA-expressing oligodendrocytes produced longer myelin sheaths on large diameter microfibers (Figs 2B, 2C, S2A and S2B) without a substantial impact on the % MBP+ oligodendrocytes (OLs) or OLs ensheathing microfibers (S2C, S2D, S2G and S2H Fig). In contrast, oligodendrocytes expressing shRNA targeting one of our candidates, the mechanosensitive cation channel Piezo1 [41] demonstrated a loss of myelin sheath elongation on the large diameter microfibers (Fig 2B and 2C).

In order to locally control sheath length, Piezo1 must be expressed in oligodendrocytes and localize to the myelin sheath. Therefore, we determined the expression and localization of Piezo1. In rats, Piezo1 expression has been shown in oligodendrocyte lineage cells: Piezo1 is present abundantly in oligodendrocyte progenitor cells (Olig2 + CC1−) and in mature oligodendrocytes (Olig2 + CC1+) during the onset of myelination [28]. Similarly, we found that Piezo1 mRNA levels were high in acutely isolated platelet derived growth factor receptor alpha (PDGRFα)-positive oligodendrocyte precursors from P6-9 mice. During the latest time points of oligodendrocyte maturation, when myelin oligodendrocyte glycoprotein (MOG) levels and 2′,3′-cyclic nucleotide 3′-phosphodiesterase (CNP) levels rose dramatically, Piezo1 levels began to diminish (S2I and S2J Fig).

To validate Piezo1 expression at the protein level, we isolated and differentiated oligodendrocytes from mice with tdTomato-tagged Piezo1 [42]. Piezo1 was detected at low levels, consistent with reports in other Piezo1-expressing cells [42,43], in approximately 40% of oligodendrocytes expressing differentiation markers BCAS1 or CNP (S2L and S2M Fig). To confirm whether Piezo1 is functional on the cell surface, we assessed calcium flux into OLs upon treatment with Piezo1-specific agonist, Yoda1 [44], at a time point when most cells exhibit highly branched morphologies and initial flat membrane formation (Fig 2D). After baseline measures of Fluo-8 calcium dye in the OLs, we treated the OLs with either Yoda1 or DMSO vehicle control. Yoda1 but not DMSO control-treated differentiated OLs demonstrated a significant fold change in intracellular calcium (Fluo-8 signal) throughout the cell (Fig 2E and 2F). To establish whether Piezo1 is localized to nascent myelin sheaths during initiation and elongation, rat oligodendrocyte microfiber cultures were immunostained when the majority of cells expressed MBP and were beginning ensheathment. Consistent with a role in diameter-sensing during initial myelin membrane formation, Piezo1 was found localized in regions of MBP+ membrane ensheathment and at the tips of ensheathment (Fig 2G and magnified Region 1) as well as in extending processes (Fig 2G magnified Region 2). Together these data established Piezo1 as a candidate for local control of myelin membrane expansion.

### Piezo1 is required for myelin sheath length scaling with large diameters in vivo

**Fig 3:**
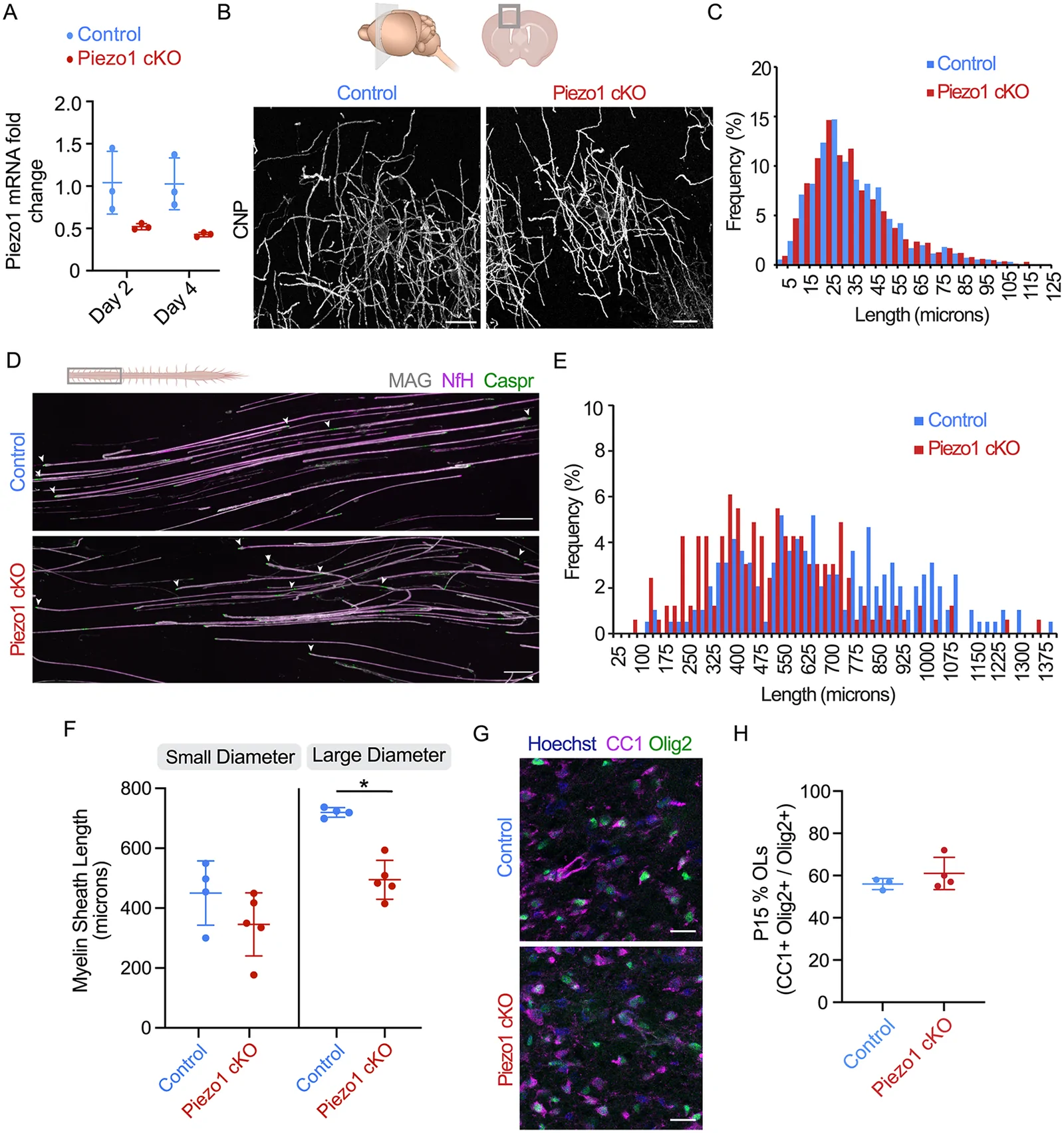
Piezo1 is important for the elongation of myelin sheaths on large diameter axons in vivo. **A)** Relative Piezo1 mRNA levels in isolated mouse OLs during differentiation measured by quantitative PCR. Relative to day 0 of floxed control mice. *p* = 0.048 *t* test between genotypes, *n* = 3 mice (cells isolated from independent animals). Bars = mean ± standard deviation. **B)** Whole OLs in layers II–III of frontal cortex sections labelled with anti-CNP. Entire OL cells can be traced to analyze the number and lengths of myelin sheaths formed. **C)** Frequency distribution of myelin sheath lengths formed in the prefrontal cortex binned by 5 micrometers, representing *n* = 4-5 mice per genotype. **D)** Immunofluorescence of teased spinal cord white matter, labelled for myelin (MAG), axonal (NfH), and paranodal (Caspr) proteins. Ends of myelin sheaths are noted with arrowheads. **E)** Frequency distribution of myelin sheath lengths (25-micrometer bins) from teased spinal cord axons. 62–142 myelin sheaths measured per mouse from *n* = 4–5 mice per genotype. **F)** Average myelin sheath length on axons smaller than 2.5 micrometers or greater than 2.5 micrometers in diameter. *p* = 0.20 for small diameter, *p = 0.001 for large diameter, Welch’s corrected *t* test between genotypes. **G)** Confocal immunofluorescence image of ventral spinal cord white matter at P15, stained for oligodendrocyte lineage marker Olig2 and mature oligodendrocyte marker CC1, alongside Hoechst for nuclei. **H)** Percent of oligodendrocyte lineage cells (Olig2+) that are mature (CC1 + Olig2+) at P15. *p* = 0.39, Welch’s corrected *t* test. The mean is shown with standard deviation. The data underlying this figure can be found under the DOIs: https://doi.org/10.6084/m9.figshare.33158513, https://doi.org/10.6084/m9.figshare.33166670, https://doi.org/10.6084/m9.figshare.33167243, https://doi.org/10.6084/m9.figshare.33169529, in the following collection: https://figshare.com/s/6e5a23b8d1765ff06f5a. The illustrations of the brain tissue section and spinal cord in Fig 3B and 3D were created in BioRender: Young, A. (2026) https://urldefense.com/v3/__https://BioRender.com/wwupi12__;!!GobTDDpD7A!MyND0zz68H0oW92eJ5sxfPUeaWlXJOr1l-NAWRmKAF_gpFiQPRpOVTzUrvzo3263GjVbtV0A83QtOq-IY_M$ and Young, A. (2026) https://urldefense.com/v3/__hhttps://BioRender.com/seeutp4__;!!GobTDDpD7A!MyND0zz68H0oW92eJ5sxfPUeaWlXJOr1l-NAWRmKAF_gpFiQPRpOVTzUrvzo3263GjVbtV0A83Qt3CNHaoU$.

To determine whether Piezo1 is critical in vivo for myelin sheath elongation, we knocked out Piezo1 during late differentiation and onset of myelination, using the CNP-Cre line [45] to drive recombination when crossed with Piezo1 floxed mice [46]. While Piezo1 antibodies work well in rat and human, they have been unsuccessful in mouse unless knocking out both Piezo1 and Piezo2 together [47]. Therefore, we first confirmed loss of Piezo1 expression in isolated mouse oligodendrocytes by mRNA quantification after differentiation. Early differentiation marker CNP mRNA was detected robustly immediately in isolated cells and rose throughout the culture (S2J Fig). Reduced Piezo1 levels in conditional knockout mice were confirmed with qPCR after 2 and 4 days of differentiation (Fig 3A). In situ hybridization of P15 ventral and lateral white matter of the spinal cord showed modest reductions in the percent of pre-OL or non-OL expressing Piezo1 mRNA, as expected (S3A, S3C and S3D Fig) based on recombination reports in other CNS regions with CNP-Cre mice [48,49]. Consistent with the percent of isolated CNP + OLs expressing Piezo1-tdTomato in culture (S2L and S2M Fig), we found approximately 40% of this population with Piezo1 mRNA in control mice. Importantly, Piezo1 cKO mice showed a similar percent of CNP+Olig2 + OLs with reduction in Piezo1 mRNA expression (S3A and S3B Fig) to what we observed with qPCR of isolated brain OLs (Fig 3A).

To establish whether Piezo1 loss impacts the number and lengths of myelin sheaths generated by oligodendrocytes in vivo, we initially assessed oligodendrocytes in the mouse prefrontal cortex at postnatal day 30. Individual oligodendrocytes and all their myelin sheaths can readily be assessed in layers II-III in the mouse cortex at this time point [50] when peak myelination has occurred, but the area is still undergoing active incorporation of new oligodendrocytes [51,52]. Thick vibratome sections were used to preserve whole oligodendrocytes. Immunostaining for the paranodal marker Caspr alongside CNP and MBP facilitated tracing whole oligodendrocytes and measuring all the connected myelin sheaths (Fig 3B). Piezo1 cKO mice had both comparable myelin sheath lengths and number of myelin sheaths formed per oligodendrocyte relative to floxed control littermates (Figs 3C and S3E–S3G).

Since the cortex has predominantly small diameter axons (below 2 micrometers [53]), we turned to the ventral and lateral spinal cord, a region of the mouse CNS with a higher proportion of large diameter axons [54]. To assess myelin sheath lengths in the densely myelinated spinal cord tracts, we used a well-established method of teasing spinal cord white matter in order to distinguish single axons and individual myelin sheaths [55]. Immunostaining for paranodal Caspr, axonal neurofilament H (NfH), and myelin associated glycoprotein (MAG), enabled us to measure individual myelin sheath length and the corresponding axon diameter (Fig 3D). Piezo1 cKO mice versus the floxed control littermates or CNP-Cre control mice showed substantially reduced myelin sheath lengths (Figs 3E and S3H-S3J). Furthermore, when comparing myelin sheath lengths formed on similar diameter ranges, i.e., small diameters or large diameter axons, Piezo1 cKO reduced the average myelin sheath lengths found on large diameter axons (Fig 3F). Importantly, since myelin sheath lengths scale with axon diameter in vivo, we verified that the axon diameter distributions were unchanged (S3K and S3L Fig). Since CNP is active in differentiating oligodendrocytes, we confirmed that CNP-Cre driven Piezo1 reduction did not impact the differentiation or density of oligodendrocytes by assessing CC1 + Olig2 + mature oligodendrocyte at postnatal day 15 in the spinal cord (Figs 3G,3H and S3M). Altogether, this mirrored our in vitro microfiber results and demonstrated Piezo1 is important in vivo for scaling myelin sheath lengths with large diameter axons.

### Piezo1 is not required to establish normal myelin thickness

**Fig 4:**
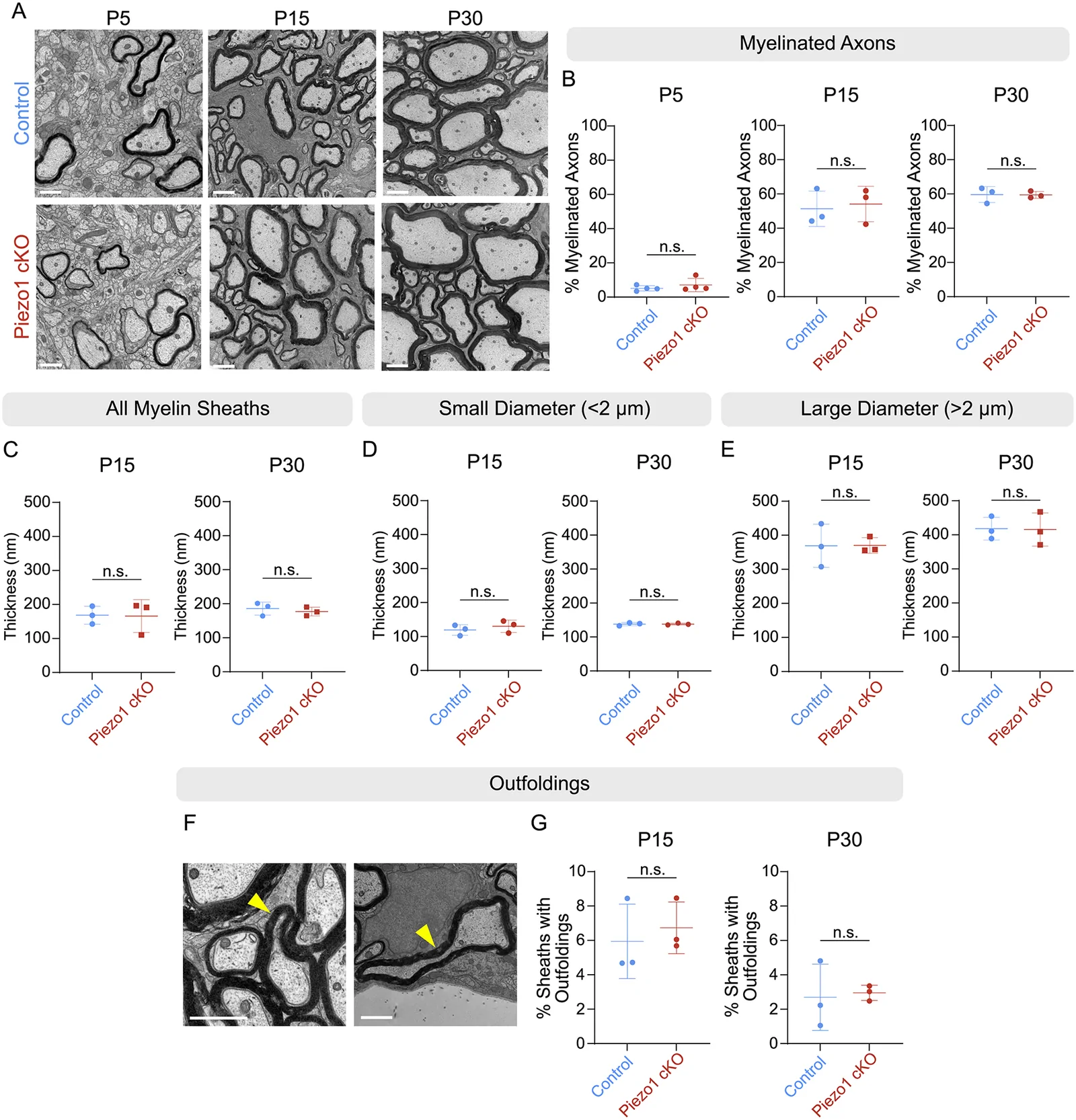
Myelin sheath onset and ultrastructure is not impacted by Piezo1 loss. **A)** Electron microscopy images of ventral cervical spinal cord of control (fl/fl) and Piezo1 cKO mice. Scale = 1 µm. **B)** The percent of myelinated axons is unchanged at P5 (*p* = 0.40), P15 (*p* = 0.38), and P30 (*p* = 0.48). *n* = 3–4 mice, >300 axons/mouse. **C)** Average myelin sheath thickness at P15 and P30, *p* = 0.47 at P15, *p* = 0.28 at P30. *n* = 3 mice, 189–247 sheaths/mouse. All *p* values comparing littermate control (Piezo1 fl/fl) and Piezo1 cKO mice using an unpaired *t t*est with Welch’s correction. **D)** Myelin sheath thickness on small diameter axons (<2 µm) at P15, *p* = 0.23, and P30, *p* = 0.49. *n* = 3 mice, 150–212 axons/mouse. **E)** Myelin sheath thickness on the large diameter axons (>2 µm) at P15, *p* = 0.49, and P30, *p* = 0.47. *n* = 3 mice, 25–46 axons/mouse. **F)** Representative images of myelin outfoldings from P15 mice. Yellow arrowheads indicate myelin outfoldings. Scale bar = 1 µm. **G)** Percent of myelin sheaths that contain outfoldings at P15, *p* = 0.40 and P30, *p* = 0.84. *n* = 3 mice, with 385–561 sheaths/mouse. Graph bars indicate mean and standard deviation. The data underlying this figure can be found under the DOIs: https://doi.org/10.6084/m9.figshare.33176519, https://doi.org/10.6084/m9.figshare.33176531, https://doi.org/10.6084/m9.figshare.33170138 in the following collection: https://figshare.com/s/6e5a23b8d1765ff06f5a.

Since the number of myelin sheath layers (i.e., thickness) is also known to scale with axon diameter [54], we set out to determine if Piezo1 is important for this process. To assess the impact on axonal ensheathment and myelin thickness after Piezo1 loss, we performed transmission electron microscopy (TEM) on ultrathin tissue sections from Piezo1 cKO mice at postnatal day 5, 15 and 30. We analyzed the cervical ventral white matter of the spinal cord (Fig 4A) because of the broad range axon diameters in this region (S4A–S4D Fig) and sheath length differences were observed in this region. Consistent with the notion that Piezo1 loss in differentiated oligodendrocytes does not impact the initiation of myelin formation, we found no difference in the percent of myelinated axons at P5, P15, or P30 (Fig 4B). At P5, during early stages of myelination, we also saw no difference in the diameter of myelinated axons (S4B Fig). There was also no difference in the distribution of unmyelinated axon diameters between littermate controls and Piezo1 cKO mice. Unmyelinated axons over 1.5 µm were rare (S4C–S4H Fig), demonstrating myelin sheath initiation on large diameter axons is unaffected.

Based on the current model of myelin sheath formation suggesting that myelin sheath lengthening is coupled to myelin thickening [56], we might expect decreased myelin sheath thickness with a myelin sheath length deficit. Interestingly, we found no differences in overall myelin sheath thickness (Figs 4C and S4E–S4L). Since Piezo1 impacted sheath lengths in a diameter-dependent manner, we predicted that Piezo1 would impact thickness in a diameter-dependent manner as well. To assess diameter-specific effects, we binned our myelin measurements into groups based on axon diameter. Neither axon diameters less than 2 µm nor greater than 2 µm had differences in myelin thickness (Figs 4D, 4E, and S4G–S4J). Together these results show that Piezo1 does not impact myelin sheath initiation or thickness, regardless of axon diameter.

In previous work, dampening oligodendrocyte intracellular calcium decreased myelin sheath lengths and increased myelin outfoldings, suggesting intracellular calcium rise is important for the directionality of myelin sheath growth [49]. Since Piezo1 is a mechanosensitive cation channel that allows calcium flux into cells, we predicted that loss of Piezo1 may also increase the number of myelin outfoldings, defined as abnormal myelin sheath outgrowth away from the axon (Fig 4F). Unexpectedly, we saw no difference in the percent of myelin sheaths with outfoldings at either developmental time point between control and Piezo1 cKO mice (Fig 4G). Other aberrant myelin, such as myelin whorls [57], and axonal morphologies indicative of degeneration [58] were scarcely observed in these samples and therefore were not quantified. Altogether, these data show that myelin sheath ultrastructure is unaffected in Piezo1 cKO mice.

## Discussion

Myelin sheath size impacts neuronal signal speed [2]. In particular, sheath length is proposed to be instrumental in coordinating signal timing for nervous system function [9–11,59]. If true, understanding how myelin sheath lengths are established and adjust their properties is critical to understanding neural networks and behavior. A combination of adaptive signals (e.g., neuronal activity), an intrinsic program (i.e., oligodendrocyte cell origin), and physical cues (e.g., axon diameter) all contribute. While electrical and biological signaling from other cells certainly can bias axon selection and adjust sheath size during myelin formation [60–64] studies have shown myelination can occur independently of neuronal activity [18,20–22,24]. Importantly, sheath lengths formed independent of neuronal activity are only slightly shorter than those in the presence neuronal activity [60,63]. Thus, these active neuronal signals do not appear to explain the striking correlation in the CNS of sheath length and thickness scaling with axon diameter [15,16,54]. Alternatively, we previously proposed that a hard-wired program in oligodendrocytes can initiate the myelination process, based on data from neuron-free cultures of oligodendrocytes on inert substrates [25]. Myelin sheath lengths can be established by oligodendrocytes, have intrinsic differences based on oligodendrocyte origin, and adapt sheath lengths in response to physical cues, such as axon diameter [25]. A combination of these intrinsic signaling pathways may converge with those from other cells and signals from neuronal activity, to establish and fine-tune myelin sheath size to the underlying axon. Until now it has remained unclear how ‘hard-wired’ myelination programs respond to axon diameter and if they regulate sheath size globally (cell-wide) or locally, on a sheath-by-sheath basis.

In this work, by using microfibers with varied diameters, we demonstrated that diameter-dependent mechanotransduction controls myelin sheath length locally, within each independent sheath (Fig 1). This is consistent with an earlier electron microscopy observation in mouse tissue where a single oligodendrocyte generated different myelin sheath thicknesses corresponding with axon diameters [37]. Our data provides direct evidence that diameter can control myelin lengths on a sheath-by-sheath level.

Here we identify the Piezo1 mechanosensitive channel as a key protein by which oligodendrocytes detect axon diameter and guide myelin sheath formation locally, in individual sheaths. Knockdown of Piezo1 in oligodendrocytes on mixed-diameter microfibers selectively affected myelin sheath length on large-diameter synthetic axons, whereas sheaths originating from the same oligodendrocytes were unaffected on smaller diameter axons. This diameter-dependent effect was recapitulated in vivo: reduction of Piezo1 in differentiated OLs shortened sheath lengths on large diameter axons in the spinal cord but not myelin sheaths formed on small diameter axons in the spinal cord nor in the cortex, a region with small diameter axons. We note that the fraction of OLs expressing Piezo1 and incomplete Cre recombination may reduce the sensitivity to detect subtle effects in the cortex; however, the consistency between the in vitro microfiber data with cortical cells and the in vivo spinal cord analysis supports a diameter-dependent role for Piezo1 in regulating myelin sheath length. The differential response to diameter suggests that Piezo1 discerns between large- and small-diameter fibers based on physical differences, such as fiber curvature. Piezo1 increases membrane permeability of cations, such as calcium, into cells upon changes in membrane tension and curvature [41,65]. Cryo-EM structural data suggests that Piezo1 induces local membrane bending in its closed-channel state; in less bent membranes, Piezo1 is flatter and in a more open conformation [66,67]. This is consistent with our data suggesting that less curvature on larger diameter fibers likely shifts the equilibrium of Piezo1 towards the open-channel state.

We propose that the role of Piezo1 in distinguishing large-diameter axons is most important for elongation during early myelin sheath formation. Our results show that Piezo1 is expressed early in differentiated oligodendrocytes then decreases with maturation (Figs 2E–2G and S2I–S2M), consistent with reports where Piezo1 expression was observed in OPCs and CC1+ oligodendrocytes in young, but not aged, rats [28]. This also aligns with observations previously reported where calcium transients and waves in myelin sheaths are most prevalent during early myelin formation then decrease over time [68,69]. Thus, we hypothesize Piezo1 contributes to these early calcium dynamics to establish diameter-dependent sheath lengths. This agrees with Auer and colleagues, who demonstrated that in vivo myelin sheath length differences are established within a 3-day window from initiation. During this early time window, myelin sheaths undergo their most rapid elongation [70], paralleling the observed expression profile of Piezo1.

Strikingly, our in vivo data demonstrates differential regulation of myelin sheath length and thickness (number of layers around an axon). Sheath lengths are shorter in Piezo1 cKO teased spinal cord preparations compared to controls (Figs 3 and S3) without any significant changes to sheath thickness (Figs 4 and S4). This data challenges the idea of inherent coupling of sheath length and thickness. Scaling of both myelin sheath length and thickness with axon diameter has been described since the late 1940s [15–17,54]. Over the years, many models of myelination have been proposed but have kept these two parameters intertwined. For example, the model proposed by Snaidero and colleagues in 2014 suggests that spiral wrapping of myelin around the axon drives lateral elongation of myelin sheaths [56]. Our data strongly supports that the mechanisms regulating myelin longitudinal growth and membrane wrapping (thickness) are independent pathways. Other studies also point to independent regulation of myelin sheath length and thickness. For example, monocular deprivation has been shown to decrease sheath length, without impacting overall sheath thickness [60]. Small reductions in MBP mRNA have been shown to decrease sheath thickness but not sheath length [71]. Alternative to the hypothesis that length and thickness are coordinately regulated, myelin sheaths could maintain the amount of membrane delivered but distribute that membrane differently. Thus, shorter myelin sheaths may be expected to have a corresponding increase in myelin layers or mistargeted myelin outfoldings. Indeed, several genetic mouse models with an observed increase in myelin outfoldings and swellings, such as oligodendrocyte-specific dampening of calcium signaling or loss of ubiquitin ligase Fbxw7, show consequences on overall myelin sheath length but not thickness [49,72]. Yet, neither outfoldings nor a change in thickness was observed in the Piezo1 cKO mice, suggesting membrane delivery and targeting may both be coordinated by Piezo1 signaling. In particular, the finding that dampening calcium in oligodendrocytes affects myelin sheath length with little evidence of an effect on thickness [49] is of special interest due to the tentative connection of Piezo1 to oligodendrocyte calcium signaling, as a cation channel. Together, this supports a connection between calcium signaling and myelin sheath lengthening but not sheath thickness during developmental myelination.

There may be common cation-initiated signaling mechanisms underlying myelin sheath growth activated by different stimuli. Reducing cation influx and/or membrane hyperpolarization via loss of cation channels impacts myelin sheath lengths [35,49,73]. For example, loss of HCN2 channels in oligodendrocytes hyperpolarizes the membrane and results in shorter myelin sheaths [73]. Global loss of the cationic mechanosensitive channel TMEM63A decreases myelin sheath growth on small diameter axons in zebrafish and mice [35]. Dampening overall calcium influx in oligodendrocytes decreases myelin sheath length [49]. Further, neuronal activity-activation of OL-expressed mGluR5 increases myelin sheath calcium-signaling and myelin sheath elongation [74]. While these diverse channels and stimuli may converge on modulating myelin sheath length, suggesting they share common downstream pathways, distinct phenotypes from their manipulation points to downstream signals that are not functionally identical. For example, dampening calcium rise results in outfoldings [55]; loss of TMEM63A results in aberrant wrapping of axons smaller than those typically myelinated as well as thinner myelin on normally myelinated axons [35], phenotypes not observed in Piezo1 cKO mice. These differences suggest that cation influx differs by channel type, activation timing, magnitude of signals, and/or precise localization, which warrants future study. Additionally, future studies investigating downstream signaling of Piezo1 in myelin sheaths will be an important avenue to understand whether diameter-dependent myelin sheath growth has shared or distinct signals to the size and ultrastructure of myelin sheaths.

It has been proposed that even small changes in myelin morphology affect axonal conduction, which can impact the precise temporal control of neuronal firing [8,60,75]. Indeed, some studies have proposed that the property of myelin sheath length could be the primary means to coordinate timing in particular neural circuits [7,8]. Direct experimental evidence demonstrating the impact on neuronal signaling when only CNS myelin sheath lengths change (e.g., without concomitant changes to thickness) are lacking. Piezo1 cKO mice display an average of 225 micrometers shorter myelin sheaths on large-diameter axons (Fig 3) without thickness differences. Thus, this work highlights a potential future avenue to experimentally uncouple the impact of length and thickness on neuronal signaling.

In this work, we address a longstanding question of how oligodendrocytes sense and scale myelin sheath lengths with axon diameter. This correlation has implications for neuronal signaling and has been observed in vivo for over 75 years without a mechanism. Our data points to a model in which oligodendrocytes locally sense axon diameter in the earliest stages of myelination through Piezo1, during a period of rapid myelin sheath elongation. Our data supports this model by demonstrating myelin sheath length is regulated at the individual myelin sheath level and by showing Piezo1 plays a critical role in myelin sheath elongation on large diameter axons. We also show that myelin sheath length and thickness can be uncoupled, as reduction in sheath elongation did not impact myelin sheath layers. This challenges existing models of myelination by demonstrating independent regulation of length and thickness and serves to further our understanding of mechanisms underlying myelination during central nervous system development.

## Materials and methods

### Ethics statement

All animal procedures were conducted in accordance with the Guide for the Care and Use of Laboratory Animals and approved by the SUNY Upstate Medical University’s institutional animal care and use committee (as per protocol no. 480).

### Primary rat oligodendrocytes

Oligodendrocyte precursor cells (OPCs) were isolated from P0-P2 Sprague-Dawley rats (Charles River). Cerebral cortices were collected and pooled from one or more litters. Meninges were removed from isolated cortices, then cortices were minced and enzymatically dissociated for 1 h at 37 °C with 1.2 U/mL papain (Worthington), 0.1 mg/mL L-cysteine (MilliporeSigma) and 0.40 mg/mL DNase I (MilliporeSigma). Mixed glial cells were plated at a density of 1.5 cortices per poly-D-lysine (PDL) coated T75 flask and cultured at 37 °C in 7.5% CO_2_ in high-glucose DMEM (Gibco), 10% fetal bovine serum (FBS, Gibco and MilliporeSigma) and 1% penicillin/streptomycin (pen/strep, Gibco). After 10 days, OPCs were enriched by mechanical shaking [76]. Shake-off cells were plated onto petri dishes for 30 m to further remove unwanted glia and non-adherent OPCs were collected for culturing.

### Mice

Piezo1 flox/flox mice, with LoxP sites flanking exons 20–23, were originally generated by the Patapoutian lab [46] and were obtained from Jackson Laboratories (RRID: IMSR_JAX:029213). To generate mice with conditional Piezo1 knockout in differentiating cells (cKO), homozygous Piezo1^fl/fl^ animals were crossed with mice with heterozygous expression Cre recombinase under the control of the 2′‐3′ cyclic nucleotide phosphodiesterase (CNP) gene [45] to generate flox control (CNP^WT/WT^; Piezo1^fl/fl^) and cKO littermates (CNP^WT/Cre^; Piezo1^fl/fl^). CNP-Cre control mice (CNP^WT/Cre^) were sourced from breeding CNP^WT/WT^ with CNP^WT/Cre^ parents. Mice were either genotyped by Transnetyx or using the following primers. CNP-Cre primers: CATAGCCTGAAGAACGAGA, GATGGGGCTTACTCTTGC, GCCTTCAAACTGTCCATCTC; Piezo1 flox primers: GCC TAG ATT CAC CTG GCT and GCT CTT AAC CAT TGA GCC ATC T.

B6;129-Piezo1^tm1.1Apat^/J were originally generated by the Patapoutian lab [42] and were obtained from Jackson Laboratories (RRID: IMSR_JAX:029214). These mice express a C-terminally tagged Piezo1-tdTomato fusion protein from the endogenous Piezo1 promotor. Mice were genotyped by Transnetyx for confirmation.

### Primary mouse oligodendrocytes

Immunopanning mouse OPCs from P6-P9 pup cerebral cortices was done using a modified method based upon [76]. After euthanasia and collecting cerebral cortices, a tail clip was taken for subsequent genotyping. Each pair of cerebral cortices was enzymatically and mechanically dissociated using a gentleMACS dissociator and the Neural Tissue Dissociation Kit P (Miltenyi). Cells were resuspended to single cell suspension in 0.2% BSA and 5 µg/mL insulin in Dulbelco’s PBS (MilliporeSigma), then negatively selected with Griffonia Simplicifolia Lectin (Vector Labs)-coated petri dishes twice for 15 m. Cell suspensions were then transferred to anti-PDGFRα (CD140a, BioLegend 135902, RRID: AB_1953328) coated petri dishes for 45 m for positive selection. Attached cells were collected. Cells were pelleted and resuspended prior to plating on a PDL-coated cell culture dish in culture medium. Medium was composed of DMEM:Neurobasal Media, B-27 alternative made in-house (as below), 5 μg/mL N-acetyl cysteine, and 10 ng/mL D-biotin, ITS supplement (MilliporeSigma), and modified Sato (100 µg/mL BSA fraction V, 60 ng/ml Progesterone, 16 µg/ml Putrescine, 40 ng/mL Tri-iodothyroxine; reagents from MilliporeSigma) and penicillin–streptomycin. The house-made B-27 (reagents from MilliporeSigma) was based upon Chen and colleagues [77], with modifications as follows: bovine serum albumin (Cat# A1470), synthetic L-carnitine hydrochloride (Cat# C0283), and synthetic (±)-α-Tocopherol (Cat# T3251). Media was supplemented with growth factors 10 ng/ml PDGF‐AA, 5 ng/ml neurotrophin 3 (NT3) (Millipore Sigma) and 10 ng/ml ciliary neurotrophic factor (CNTF) (Peprotech) and 2.05 µg/mL forskolin (Cayman Chemical 11018). Cells were cultured at 37 °C and 7.5% CO_2_ with a half media change every other day with new growth factors.

*qPCR*

Piezo1 expression analysis and confirmation of knockout in oligodendrocytes was performed by qPCR. Oligodendrocytes were isolated from CNP+/Cre, CNP+/Cre; Piezo1 fl/fl, and Piezo1 fl/fl mice. Isolated mouse OPCs were divided evenly between Trizol lysis (for Day 0) and PDL-coated cell culture plates for collection on days 2, 4, 6, 8, and/or 10. RNA was extracted from cells with Trizol followed by chloroform extraction using a Monarch Total RNA Miniprep Kit. DNase IXT (New England Biolabs) was used for in-column DNase treatment. cDNA generated with the LunaScript RT SuperMix Kit (New England Biolabs). IDT PrimeTime Standard qPCR assay and qPCR probe and primer sets were used for qPCR. Primer and probe sets were as follows:

CNP: AGAGAGCAGAGATGGACAGT, AATTCTGTGACTACGGGAAGG,/5Cy5/AGCAGGAGG/TAO/TGGTGAAGAGATCGTA/3IAbRQSp/
Piezo1: CATGCGTTGCCACTCCT, GGCTGTACCTACCTGACTTCT,/5SUN/CCTTATCAG/ZEN/TGACTTCCTCCTGCTGC/3IABkFQ/
GAPDH: GTGGAGTCATACTGGAACATGTAG, AATGGTGAAGGTCGGTGTG,/56-FAM/TGCAAATGG/ZEN/CAGCCCTGGTG/3IABkFQ/
MOG: AGTCCGATGGAGATTCTCTACT, CACTTGTGCCTACGATCCTC,/5Cy5/CACGAAGTT/TAO/TTCCTCTCAGTCTGTGCT/3IAbRQSp/

Multiplexed qPCR was performed on a BioRad CFX Opus. All Ct values were normalized against GAPDH values, to obtain ΔCt. For expression across time, ΔΔCt was normalized against expression at time of cell isolation. Fold change expression between Piezo1 cKO and floxed control mouse cells were calculated with 2^−(ΔΔCt)^ relative to floxed controls at Day 0.

### Mouse primary culture immunofluorescence

Cells were fixed at day 2 and 4 in culture with 4% formaldehyde in PBS, followed by PBS washes, and permeabilization in 0.1% TritonX-100 in PBS. Primary antibodies were diluted in PBS and incubated overnight at 4 °C, as follows: rabbit anti-RFP 1:100 (Rockland 600-401-379, RRID: AB_2209751), mouse anti-BCAS1 1:500 (Synaptic Systems 445,011, RRID: AB_2925006), mouse anti-CNP 1:300 (BioLegend 836404; RRID: AB_2566639). Secondary Alexa Fluor 488, 555, and 633-conjugated secondary antibodies (Invitrogen) were diluted 1:1000 in PBS and incubated for 1 h. Cells were washed with PBS then stained with 5 µg/mL Hoechst (Caymen Chemical 15547) for 10 min. Cells were mounted with Fluoromount G (Southern Biotech). All experiments were conducted with negative staining control cells from the same date to control for non-specific fluorescence.

Confocal 1,024 × 1,024 images were acquired on a Leica SP8 microscope with 40×/1.10 water objective, using 0.42 µm z-steps. Ten random areas were imaged across each coverslip. Image acquisition (laser intensity, gain, etc.) and processing settings (e.g., brightness, contrast) were optimized using random fields of view from negative staining control and Piezo1-tdTomato cells. The same settings were used uniformly across all images from a single experiment.

### Electrospun fiber cultures

Electrospun microfibers composed of poly-L-lactic acid were synthesized as custom, parallel-aligned fibers and suspended by fitting into 12-well plate inserts by The Electrospinning Company. Specific fiber diameter ranges of 1–2 µm, and 2–4 µm were used as reported previously [24]. Mixed fiber diameter microfiber scaffolds spanning fiber diameters between 0.7 and 6 micrometers distributed across each scaffold were generated and verified with scanning electron microscopy by The Electrospinning Company. Microfiber scaffolds were soaked in 70% ethanol for 10 m, washed, then coated with PDL. Rat OPCs were added at 30,000–35,000 per scaffold in myelin medium [36]. Medium was changed every 2–3 days. Myelin medium was as follows: 50:50 high-glucose DMEM:Neurobasal Media, B27 (Gibco) or a B-27 alternative (as above), 5 μg/mL N-acetyl cysteine, and 10 ng/mL D-biotin, ITS supplement (MilliporeSigma), and modified Sato (as above but with 400 ng/mL Tri-iodothyroxine, 400 ng/mL L-Thyroxine; reagents from MilliporeSigma), and penicillin–streptomycin.

### Inducible knockdown of gene expression

Rat OPCs were transduced with SMARTvector inducible lentiviruses (TU at least 10^7^/mL) for shRNA-mediated knockdown generated by Horizon Discovery. shRNA sequences used are as follows: Piezo1 shRNA: 1- AAGTACGACCTGGTGCAAC, 2 – TCACGGGCATCTACGTCAA, 3 – TGCTGTGCCTCACGTGTT and the supplemental table (S1 Table). These Tet-On 3G inducible viruses were generated to express shRNA targets using mouse CMV promoter, including turboGFP as a reporter of expression. Non-targeting control shRNA (VSC6584, HorizonDiscovery) was used to account for non-specific effects of lentiviral shRNA and turboGFP. Pooled lentiviruses generated against three target shRNA sequences were used. Prior to lentiviral transduction, 200,000 rat OPCs were plated into each well of a PDL-coated 6-well plate with proliferation medium. After 24 h cells were given fresh proliferation medium and incubated overnight with lentivirus at 5 MOI, as optimized based upon turboEGFP expression and cell viability after 72 h. Proliferation medium was as follows: high-glucose DMEM (Gibco), ITS (Sigma), and modified Sato (as above), pen–strep, and 0.5% FBS with fresh 10 ng/ml PDGFa and 10 ng/ml bFGF (Peprotech). After overnight incubation with viruses, cells were washed with D-PBS (Sigma), dissociated from the plates with TrypLE (Gibco), spun down and resuspended in myelin medium, then plated at 35,000 OPCs per 12-well microfiber insert.

### Microfiber culture immunofluorescence and analysis

After 14 days in culture, cells were fixed in 4% formaldehyde/PBS, followed by PBS washes, and permeabilization in 0.1% TritonX-100 in PBS. Primary antibodies were diluted in PBS and incubated overnight at 4 °C. Primary antibodies were: rat anti-myelin basic protein 1:250 (Biorad MCA409S, RRID: AB_325004), chicken anti-GFP 1:500 (Abcam ab13970, RRID: AB_300798). Cells were washed in PBS and incubated 1 h with Alexa Fluor 488, 568, or 647 conjugated secondary antibodies (Invitrogen), used at 1:1,000. After three PBS washes, cells were stained with 5 µg/mL Hoechst (Sigma-Aldrich), washed again and mounted with Fluoromount G (Southern Biotech) with cover glass onto glass slides.

Images were obtained on a Leica SP8 confocal scanning microscope, with a 40× oil/NA1.3 objective. Confocal stacks of 0.35 µm z-steps were taken at 1,024 × 1,024. At least 10 random areas containing MBP positive cells were imaged across each coverslip. All settings were kept the same within each replicate experiment and images were blinded.

Fiji ImageJ was used to analyze myelination. For comparisons of myelin sheaths on mixed diameter fibers, it was not possible to blind the experimenter from the fiber diameter. Sheaths were defined as >4.5 micrometer-long continuous MBP positive membranes fully surrounding a microfiber as assessed using the 0.35 µm z-series. Tubes were traced to measure the length and a perpendicular line was drawn to measure the corresponding fiber diameter. The frequency of sheath lengths was calculated for 5 µm bins. Frequencies from at least three independent experiments were generated and plotted as a frequency distribution. The sheath lengths were determined to be log gaussian distributions, therefore mean log lengths were used for statistics. Binned log lengths (0.1 bin size) were also used to generate frequency distribution plots. For each oligodendrocyte analyzed, myelin sheaths formed on different caliber fibers were measured from the same cell. At least three independent experiments were cells pooled from different rat litters on different days. A minimum number of sheaths were measured for microfiber experiments, as indicated in figure legends.

### Calcium imaging

Ibidi 35 mm No 1.5 polymer coverslip dishes (Cat #81156) were PDL coated. 165,000 cells were plated across the entire surface area in differentiation media as follows: high-glucose DMEM media, 0.25% FBS (MilliporeSigma or Gibco), ITS supplement (MilliporeSigma I1884-1VL), modified SATO (as above), GlutaMAX (Gibco), Sodium pyruvate (Sigma-Aldrich) and pen/strep (Gibco).

Oligodendrocytes after 2 days of differentiation were labelled with calcium and membrane dyes. 1:10,000 Fluo-8 (AAT Bioquest 21080) diluted in MEM (Sigma-Aldrich) was incubated 30 min at 37 °C then 30 min at room temperature, followed by 1:5,000 CellMask Deep Red Plasma Membrane (Invitrogen C10046) in MEM for 5 min at 37 °C. Cells were washed with MEM and FluoroBrite DMEM (Gibco A1896701), then switched to photostable live imaging differentiation medium: FluoroBrite DMEM supplemented as above.

Cells were imaged at 37 °C with humidified 5% CO_2_ on a Leica SP8 confocal microscope with a 25×/0.95 water objective. 16-bit images were acquired with 512 × 512 format, speed 700 Hz and pinhole opened to 10.74 with 5.29 µm z-steps. Live data mode was used to program acquisition of 3.75–5 min of baseline with images every 2 sec, a pause for drug addition, followed by 3.75–10 min of treatment measurements every 2 s. Final concentrations were: DMSO 1 µL/mL (Invitrogen) Yoda1 5 µM in DMSO (Cayman Chemical 21904 or Sigma-Adrich SML1558). All treatments were followed by imaging 1–5 min (2 s intervals) upon 10 µM ionomycin (Cayman Chemical 11932) treatment to verify a Fluo-8 signal in response to calcium rise.

Images were blinded using the FIJI ImageJ blind analysis. Membrane dye was used to determine individual oligodendrocyte cell boundaries, regions of interest (ROI). Background measurements were taken in areas without cells. Background corrected fluorescence intensities calculated as such: ROI integrated density − (ROI area*mean of background). Then change in fluorescence intensity (Δ*F*/*F*_0_) was calculated: (*F*_*t*_ − *F*_0_)/*F*_0_ where *F*_*t*_ is fluorescence at time *t* and *F*_0_ is the average fluorescence intensity for the entirety of baseline. Amplitude was defined as the maximum fluorescence intensity during the first 3 min of treatment period. Statistics were run on amplitude of biological replicates from 5 independent rat cell isolations.

### CNS tissue acquisition for teased spinal cords, vibratome and cryosections

Mice were euthanized by cardiac perfusion fixation under anesthesia by flushing with PBS followed by 4% formaldehyde in PBS. Brain and spinal cord tissue was isolated immediately after formaldehyde perfusion fixation of mice. Tissue was post-fixed in 4% formaldehyde in PBS either for 30 min or overnight at 4 °C (see below).

### In situ *hybridization of tissue cryosections and associated analysis*

Overnight post-fixed and PBS washed spinal cord tissue was cryoprotected with 30% sucrose, then flash frozen in dry ice-cooled 2-methylbutane and stored at −80 °C. Cervical spinal cords were cut coronally with a Leica cryostat to generate 10 μm sections mounted onto Superfrost Plus slides (Fisherbrand). A minimum of 3–6 tissue sections per mouse a minimum of 40 μm apart were used for RNAScope in situ hybridization, following Multiplex Fluorescent Reagent Kit v2 (ACDbio) manufacturer protocols, with TSA Vivid 520 (for C1), TSA Vivid 570 (for C2), TSA Vivid 650 (for C3) dyes and the following probes: Piezo1 C1 probe (Mm-Piezo1-O1 Cat# 500511), Olig2 C2 probe (Mm-Olig2-C2, Cat# 447091-C2), and CNPase C3 probe (Mm-Cnp-C3, Cat# 472241-C3). Non-specific binding was determined with included negative control C1, C2, and C3 probes and the same mouse tissue, to determine background signal for establishing imaging settings.

Images were acquired on a Leica SP8 confocal with a 40×/NA1.1 water objective. Blinded images were acquired at 1,024 × 1,024, and z-steps of 0.42 µm with identical imaging settings across all tissue sections. Two-three images were acquired in both the lateral and ventral white matter of each spinal cord section.

Images were analyzed with a previously described RNAScope automated analysis pipeline using QuPath software [78,79]. Briefly, DAPI staining was used for nucleus segmentation plus a 3-micrometer expanded boundary was used to set the cell area for probe quantification using the ‘Cell Detection’ module. The automated detection of mRNA puncta was based on intensity thresholds. Positive cells were determined for each probe set. Due to the diffuse expression of CNP and Olig2 in cervical spinal cord tissue, positive cells were considered positive when they had puncta within their nucleus. Piezo1 positive cells were counted when any puncta above intensity thresholds were detected within the nucleus and surrounding 3-micrometer cell boundary. The percent of Piezo1 + cells that were considered non-OLs (nuclei negative for both Olig2 and CNP), pre-OLs (nuclei Olig2 + CNP−), and differentiated OLs (nuclei Olig2 + CNP+) was calculated per tissue section (technical replicate). Technical replicates were tested with Grubbs outlier analysis in GraphPad Prism. One tissue section was identified by this analysis and excluded. The technical replicate values were averaged per mouse. Each mouse was considered a biological replicate and unblinded after averages were determined.

### Immunofluorescence of tissue cryosections and differentiation analysis

Cryosections 10–15 μm thick (prepared as outlined above) were blocked in 10% donkey serum and 0.2% TritonX-100 in PBS. Primary antibodies were diluted in blocking solution and incubated overnight at 4 °C. Primary antibodies were: mouse anti-CC1 1:500 (Abcam Ab16794, RRID: AB_443473), goat anti-Olig2 1:500 (RnD AF2418, RRID: AB_2157554) and rat anti-MBP 1:500 (BioRad MCA409S, RRID: AB_32500). Sections were washed with PBS, followed by species-specific Alexa Fluor 488, 555, or 633 antibodies 1:1000 in blocking solution for 1 h. Cryosections were washed with PBS, stained with Hoechst, and mounted with Fluoromount G.

Images were obtained on a Leica SP8 confocal scanning microscope with a 40× water/NA1.1 objective. Stacks of 1 µm z-steps were taken at 1,024 × 1,024. At least two sections with a minimum of 60 µm spacing were imaged. For each tissue section, four images were acquired (two lateral, two ventral white matter). The same settings were universally applied within each replicate experiment and images were blinded. Cells that were positive for Hoechst, Olig2, and CC1 were counted from max projected images in Fiji ImageJ, using synchronize windows and the multi-point tool. Hoechst, CC1 and Olig2 positive cells from the total number Hoechst and Olig2 positive cells were calculated to assess % differentiated oligodendrocytes.

### Immunofluorescence and analysis of cortical brain sections

Cortical brain tissue preparation and analysis was done with minor modifications as in [50,71]. Overnight post-fixed and PBS washed tissue was embedded in 2% low melting point agarose in PBS. The medial prefrontal cortex was sectioned coronally in 150 μm slices on a Leica VT1000 vibratome. The prefrontal cortex slices used were between bregma 1.3 and 1.9 mm in the infralimbic and prelimbic areas. Free-floating sections were processed for antigen retrieval by 20 m incubation at 95 °C in 0.05% Tween20, 10 mM tri-sodium citrate (pH 6.0). Sections were blocked for 3 h in 10% goat serum and 0.25% TritonX-100 in PBS. Primary antibodies, diluted in blocking solution, were incubated rocking at 4 °C for 24 h. Primary antibodies were: anti-CNPase 1:2,000 (Sigma C5922, RRID: AB_476854), anti-MBP 1:250 (BioRad MCA409S RRID: AB_32500), anti-Caspr 1:250 (Abcam ab34151, RRID: AB_869934). Sections were washed in PBS followed by 4 h incubation with 1:1,000 diluted Alexa Fluor 488, 568, or 647 conjugated antibodies in blocking solution. Nuclei were stained with Hoechst followed by mounting sections onto slides with Fluoromount G.

Images were obtained with blinded samples on a Leica SP8 confocal scanning microscope, with 40× oil/NA1.3, and 40× oil/NA1.25 objectives. Stacks of 1 µm z-steps were taken at 1,024 × 1,024 resolution. Eight random fields of view (290 µm x 290 µm) of the medial prefrontal cortex layer II/III were imaged per mouse. Settings were kept the same across all brain slice images, and images were kept blinded.

Three-dimensional analysis of myelin sheath lengths was performed using the simple neurite tracer plugin [80]. Myelin sheaths were analyzed for CNP and MBP positive oligodendrocytes. Only myelin sheaths and cells where all sheaths were contained within the stack were quantified. This was assessed by following each CNP positive process from the cell body through the entire z-stack. The lengths of CNP-positive segments flanked by paranodal Caspr bands were measured. The sheath lengths were log normal, therefore both the frequency of binned raw sheath lengths as well as binned log sheath lengths were used. For statistics, a two-tailed *t* test with Welch’s correction was used to compare the average log sheath length, with individual mice as the biological replicates. Sample sizes were based on prior publications and power calculations, with power 0.8, alpha 0.05, and a 10-micrometer effect size [50,71].

### Teased spinal cord preparation and immunofluorescence

Cervical and thoracic spinal cord regions from blinded samples were post-fixed 30 m, followed by short-term storage up to 48 h in PBS at 4 °C. Strips of ventral white matter were teased onto SuperFrost Plus slides with acupuncture needles and immunostained following established procedures [55]. Briefly, after 1 h of permeabilization with 3% normal donkey serum, 2% bovine serum albumin, 0.1% Triton X-100 in PBS, primary antibodies were diluted in this buffer overnight. Primary antibodies were: neurofilament (NfH) 1:1000 (BioLegend 822601, RRID: AB_2564859), Caspr 1:500 (Abcam ab34151, RRID: AB_869934), MAG 1:100 (Santa Cruz Biotechnology sc-9544, RRID: AB_670102 and R and D Systems AF538, RRID: AB_355423). After washes, secondary Alexa Fluor antibodies were incubated in the same buffer for 1h, followed by additional washes and mounting with FluoromountG and cover glass.

Images were obtained on a Nikon Eclipse Ti2 with a Plan Apo 20×/NA0.8 objective. Random areas were imaged as tiled 0.5 µm z-step stacks at 2,048 × 2,048. Tiled images from blinded samples were stitched with the Image J pairwise or grid collection stitching plugin [81] to assure inclusion of sheaths greater than 1,000 µm in length. Myelin sheaths were measured by two individuals, measuring lengths along NfH and MAG positive myelinated axons from paranode to paranode (Caspr band to Caspr band). The axon diameters were assessed by perpendicular measurements acquired at approximately 10 µm from the paranodes. Myelin sheath lengths were both averaged per mouse as well as plotted as frequency of 25 µm binned lengths. The average and distribution of lengths were also binned into sheaths formed on axons above or below 2.5 µm in diameter. Statistics were conducted on average lengths, with individual mice considered the biological replicate. Sample sizes were based on prior publications and power calculations, with power 0.8, alpha 0.05, and a 120 µm effect size [55,71].

### Transmission electron microscopy

Mice were anesthetized and cardiac perfusion fixed at postnatal days 5, 15, and 30. Mice were perfused with 0.1 M phosphate buffer for 2–3 m then a 4% PFA/2.5% Glutaraldehyde mix in 0.1 M phosphate buffer for 10 m at 1 mL/min. The spinal cord was dissected out, meninges removed, sub-dissected into 1 mm pieces, then post-fixed overnight at 4 °C. Tissue was washed in phosphate buffer, further fixed in 1% OsO_4_ with 1.5% potassium ferrocyanide, then dehydrated with 10-minute ethanol wash series (25% ×1, 50% ×2, 70% 2×, 90% 2×, 100% ×4). Samples were rinsed with acetone, followed by infiltration with a series of transitional solvent/Epon 812 mixtures on a rotator, as follows: 2 h in a 2:1 acetone:Epon 812 mix, overnight in 1:1, 2 h in 1:3 acetone:Epon 812, then pure Epon 812 four times for a minimum of 1 h each. Sample were cured at 60 °C. Ultrathin sectioning and TEM were performed at the SUNY Upstate Medical University TEM Core. Cervical spinal cord samples were cut into 70–90 nm sections using a Leica microsystems EM UC7 ultramicrotome and Diatome diamond knife. Sections were post-stained in UranyLess (Electron Microscopy Sciences), washed in distilled water, lead citrate stained (Electron Microscopy Sciences) and washed again in distilled water. Ventral white matter images adjacent to the median fissure were imaged with a Jeol JEM-1400 series 120 kV TEM operated at 80 kV and a Gatan Orius SC-1000 CCD camera.

### Transmission electron microscopy analysis

Quantification was performed blinded using FIJI/ImageJ by two individuals. Axon diameter was determined by measuring the perimeter of the axon, then dividing by pi. Myelin sheath thickness was determined by averaging 5–10 measurements of the electron dense compact myelin layers, excluding any non-compacted areas (e.g., the inner tongue or splitting in the layers). Thickness of myelin was determined from 4–5 non-adjacent fields of view in the ventral white matter of the cervical spinal cord, yielding ~200 myelinated axons/mouse. Replicates were individual mice, with sample size based on calculations with power >0.8, alpha 0.05, and effect size of 50 nm on small diameter and 70 nm on large diameter axons.

For percentage of myelinated axons at P15 and P30, axons from the 4–5 fields of view were categorized as either unmyelinated or myelinated, which includes ensheathed axons without electron dense compacted myelin (>300 axons/mouse). For percentage of myelinated axons at P5, myelinated axons were determined by visible electron dense, compact membrane layers surrounding the axon. Unmyelinated axons <1μm diameter which lacked compact myelin were counted but perimeters were not measured (>1,000 axons/mouse). Perimeters of all axons ≥1μm diameter as well as any myelinated axon <1 μm diameter were measured (yielding ~100 axons/mouse).

For percent of myelin sheaths with outfoldings, myelin sheaths from 9 to 11 fields of view/mouse were counted, yielding 385–561 sheaths/mouse. Outfoldings were defined as myelin membranes that extend away from the axon.

### Statistics

Error bars presented are the standard deviation to show the variation. Statistical analysis was done in GraphPad Prism. Data was tested for normality using a Shapiro Wilks test. For two groups, unpaired two-tailed *t*-Tests with Welch’s correction were used. For comparisons of 3 groups, one-way Anova was used. Biological replicates and sample size was determined prior to experimentation using G*power with anticipated effect sizes from data generated in prior publications, as described in each section above and/or figure legends.

## Supporting information

S1 Fig Mixed microfiber diameters and log sheath lengths. **A)** Microfiber diameters observed from all mixed diameter cultures. The frequency distribution shows all ensheathed microfiber diameters binned in 0.5-micrometer increments. **B)** The average microfiber diameter per experiment. All fiber diameters as well as the diameters binned as “small” or “large”. **C)** The average log length of myelin sheaths formed in different microfiber cultures. *n* = 3–4 independent oligodendrocyte microfiber cultures. Bars = mean ± standard deviation. The data underlying this figure can be found under the DOIs: https://doi.org/10.6084/m9.figshare.33116207, https://doi.org/10.6084/m9.figshare.33116273, in the following collection: https://figshare.com/s/6e5a23b8d1765ff06f5a.
(PDF)

S2 Fig Knockdown in microfiber cultures and expression of Piezo1 mRNA in the mouse oligodendrocyte lineage. **A)** The average log length of myelin sheaths formed in the mixed microfiber cultures treated with inducible control shRNA or Piezo1 shRNA, binned into myelin sheaths formed on small diameter (<2.5 micrometer) or large diameter (>2.5 micrometer) fibers. **B)** Microfiber diameters observed in knockdown culture experiments. The average microfiber diameter per experiment. Diameters binned as “small” or “large”. *n* = 3–4 independent oligodendrocyte microfiber cultures. **C)** OL differentiation (% MBP+ cells) relative to control shRNA treated conditions in knockdown microfiber experiments. **D)** Proportion of MBP + OLs wrapping microfibers relative to control shRNA-treated conditions. *n* = 3 independent oligodendrocyte cultures (different pooled rat litters) on microfibers. **E)** The average log length of myelin sheaths formed in the mixed microfiber cultures on small diameter (<2.5 micrometer) fibers. OLs were induced to express control shRNA or shRNA targeting the other indicated candidates after differentiation (MBP expression). *n* = 3 independent oligodendrocyte cultures (different pooled rat litters) on microfibers. Any candidate with < 3 datapoints, indicates an insufficient number of myelinating cells in that condition/replicate. One-way ANOVA compared to shControl, *p* = 0.77. **F)** The average log length of myelin sheaths formed on large diameter (>2.5 micrometer) binned from the mixed microfiber cultures. One-way ANOVA compared to shControl, *p* = 0.85. **G)** OL differentiation relative to control shRNA treated OLs. **H)** MBP + OLs wrapping microfibers relative to control shRNA-treated OLs. *n* = independent OL cultures. **I)** Quantitative PCR of mouse oligodendrocyte lineage cells acutely isolated (Day 0) through differentiation in culture (up to Day 10 in culture). mRNA expression levels are shown relative to Day 0 (at time of PDGFRα+ mouse cell isolation) after normalization to housekeeping gene GAPDH. CNP and MOG levels rise dramatically and Piezo1 mRNA levels decrease after 8 days in culture. **J)** Delta Ct values (amplification cycle relative to GAPDH) across time points for three mice. CNP levels are already robustly present but increase further when MOG levels rapidly rise. Piezo1 mRNA levels are relatively low and decrease further in the last two days in culture. *n* = cells isolations from 3 different mice. **K)** Confocal images from primary mouse oligodendrocytes stained for OPC marker (NG2) or tdTomato (anti-RFP) from negative control mice or tdTomato-tagged endogenous Piezo1. **L)** Confocal images of primary mouse oligodendrocytes stained for nuclei (Hoechst), differerentiation markers BCAS1 (top) or CNP (bottom) as well as anti-RFP to detect tdTomato-tagged endogeneous Piezo1. Scale bars = 25 µm. **M)** Percent of BCAS1 (top) or CNP (bottom) cells that are tdTomato+ on days 2 and 4 in vitro. *n* = cell isolations from 3–7 mice (ran side by side with OLs isolated from tdTomato negative mice). Bars = mean ± standard deviation. The data underlying this figure can be found under the following DOIs: https://doi.org/10.6084/m9.figshare.33117494, https://doi.org/10.6084/m9.figshare.33117479, https://doi.org/10.6084/m9.figshare.33127754 in the following collection: https://figshare.com/s/3ec75d99d536bf381076.
(PDF)

S3 Fig Piezo1 is important for the elongation of myelin sheaths on large diameter axons in vivo. **A)** RNAScope in situ hybridization for indicated mRNA in P15 cervical ventral and lateral white matter with nuclei (DAPI) counterstain. Outlines indicate CNP + OL cells and arrowheads indicate the Piezo1 + puncta. Scale = 10 µm. Magnified images show examples of CNP+ cells outlined in orange. **B)** The fraction of all OLs (defined as nuclei associated with CNP+Olig2 + puncta) also containing Piezo1 mRNA puncta. *n* = 3 mice of each genotype, with 2–5 tissue sections analyzed per mouse (> 533 total CNP+ cells). **C)** Pre-OLs (defined as nuclei associated with Olig2 + puncta but no CNP puncta) also containing Piezo1 mRNA puncta. *n* = 3 mice of each genotype, with 2–5 tissue sections analyzed per mouse (> 459 Olig2 only cells). **D)** Non-OLs (defined as nuclei without associated Olig2 or CNP puncta) also containing Piezo1 mRNA puncta. *n* = 3 mice of each genotype, with 2–5 tissue sections analyzed per mouse (> 253 Olig2-CNP- cells). **E)** The average number of myelin sheaths formed by single OLs in the mouse frontal cortex, layers II-III at P30. *p* = 0.28, Welch’s corrected *t* test, *n* = 4–5 mice per genotype. **F)** Number of myelin sheaths formed by single OLs from all the mice. **G)** Log gaussian curve showing the distribution of myelin sheath lengths (log length) in the mouse frontal cortex at P30. *p* = 0.56 Welch’s corrected *t* test of mean log lengths (*n* = 4–5 mice). **H)** Log gaussian curve showing the distribution of myelin sheath lengths (log length) on teased spinal cord axons at P30, with a shift in sheath length distributions. **I)** The distribution of myelin sheath lengths in the mouse spinal cord from Piezo1 CKO mice, alongside both control groups: floxed control littermates and CNP-Cre control mice. Distribution of all myelin sheath lengths. **J)** Mean sheath lengths per mouse at P30 on all axon diameters as well as those binned by axon diameters less than or greater than 2.5 micrometers. Piezo1 cKO mice compared against both floxed littermate controls (Control) or CNP-Cre controls, *p* = 0.002 for all axons, *p* = 0.33 for small diameter, *p* = 0.0001 for large diameter, one-way ANOVA. *n* = 3–5 mice per genotype, at least 62 myelin sheaths measured per mouse. **K)** Distribution of the large diameter axons with measured myelin sheath lengths from teased spinal cords from 4 to 5 mice measured in D–F. **L)** Mean axon diameter per mouse. Measurements taken from all spinal cord axons measured in Fig 3. *p* = 0.30, Welch’s corrected two-tailed *t* test. **M)** Density of all immature (CC1-) and mature (CC1+) oligodendrocyte lineage cells at P15 in the mouse spinal cord. *n* = 3–4 mice per genotype, *p* = 0.39 and *p* = 0.49, Welch’s corrected *t* test. Bars = mean ± standard deviation. The data underlying this figure can be found under the following DOIs: https://doi.org/10.6084/m9.figshare.33166670, https://doi.org/10.6084/m9.figshare.33167243, https://doi.org/10.6084/m9.figshare.33169529, https://doi.org/10.6084/m9.figshare.33158348, https://doi.org/10.6084/m9.figshare.33167006 in the following collection: https://figshare.com/s/3ec75d99d536bf381076.
(PDF)

S4 Fig Myelin ultrastructural data: calculated g-ratio, individual thickness measurements, and diameter distributions from myelinated and unmyelinated axons. **A)** Distribution of myelinated axon diameter by mouse at P5, P15 and P30. **B)** Average diameter of myelinated axons at P5 (*t* test with Welch’s correction, *p* = 0.99). **C)** Frequency distribution of unmyelinated axon diameters at P15 and P30. **D)** Distribution of unmyelinated axon diameter by mouse at P15 and P30. **E–H)** Equivalent g-ratios for thickness data. g-ratios were calculated from the axon perimeter measurements and the thickness of the myelin sheath (g-ratio = axon diameter/ (axon diameter + (thickness x 2))). **E,F)** Average g-ratio at P15 (*t* test with Welch’s correction, *p* = 0.31) and P30 (*t* test with Welch’s correction, *p* = 0.89). **G,H)** Scatter plot of calculated g-ratio at P15 (simple linear regression, *R*^2^ = 0.01) and P30 (simple linear regression, *R*^2^ = 0.28). **I,J)** Scatter plot of myelin thickness measurements at P15 (simple linear regression, *R*^2^ = 0.79) and P30 (simple linear regression, *R*^2^ = 0.84). **K,L)** Average sheath thickness including CNP-Cre controls at P15 (cKO vs. CNP-Cre, *p* = 0.87) and P30 (*p* = 0.62). *p* values from *t*-tests with Welch’s correction between two genotypes. The data underlying this figure can be found under the following DOIs: in the following collection: https://doi.org/10.6084/m9.figshare.33176519, https://doi.org/10.6084/m9.figshare.33176531, https://doi.org/10.6084/m9.figshare.33176540 https://figshare.com/s/3ec75d99d536bf381076.
(PDF)

S1 Table shRNA Target Sequences. (DOCX)

## Abbreviations

CNP: 2',3'-cyclic nucleotide 3′-phosphodiesterase
CNS: central nervous system
CNTF: ciliary neurotrophic factor
MAG: myelin associated glycoprotein
MBP: myelin basic protein
MOG: myelin oligodendrocyte glycoprotein
NfH: axonal neurofilament heavy chain
OPCs: oligodendrocyte precursor cells
PDL: poly-D-lysine
ROI: regions of interest
TEM: transmission electron microscopy
