# Supplementary material for: Myelin sheath lengths in the central nervous system scale to axon diameter via oligodendroglial Piezo1
Source: PLoS Biol. 2026 Sep 21;24(9):e3003992. doi: 10.1371/journal.pbio.3003992 (PMC13592707; doi:10.1371/journal.pbio.3003992)
Supplement: Supplementary file 1 [file pbio.3003992.s001.pdf]

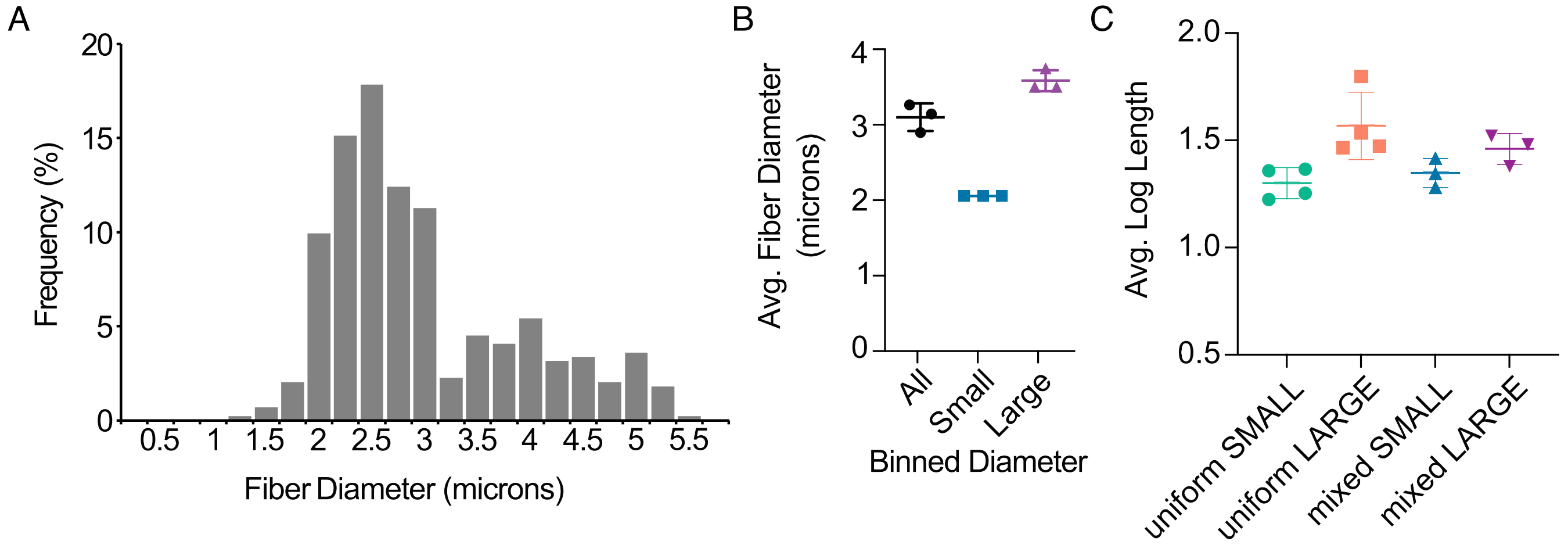

A
20
15
10
5
0
Frequency (%)
0.5
1
1.5
2
2.5
3
3.5
4
4.5
5
5.5
Fiber Diameter (microns)
B
4
3
2
1
0
Avg. Fiber Diameter (microns)
All
Small
Large
Binned Diameter
C
2.0
1.5
1.0
0.5
Avg. Log Length
uniform SMALL
uniform LARGE
mixed SMALL
mixed LARGE
