# Supplementary material for: Myelin sheath lengths in the central nervous system scale to axon diameter via oligodendroglial Piezo1
Source: PLoS Biol. 2026 Sep 21;24(9):e3003992. doi: 10.1371/journal.pbio.3003992 (PMC13592707; doi:10.1371/journal.pbio.3003992)
Supplement: Supplementary file 2 [file pbio.3003992.s002.pdf]

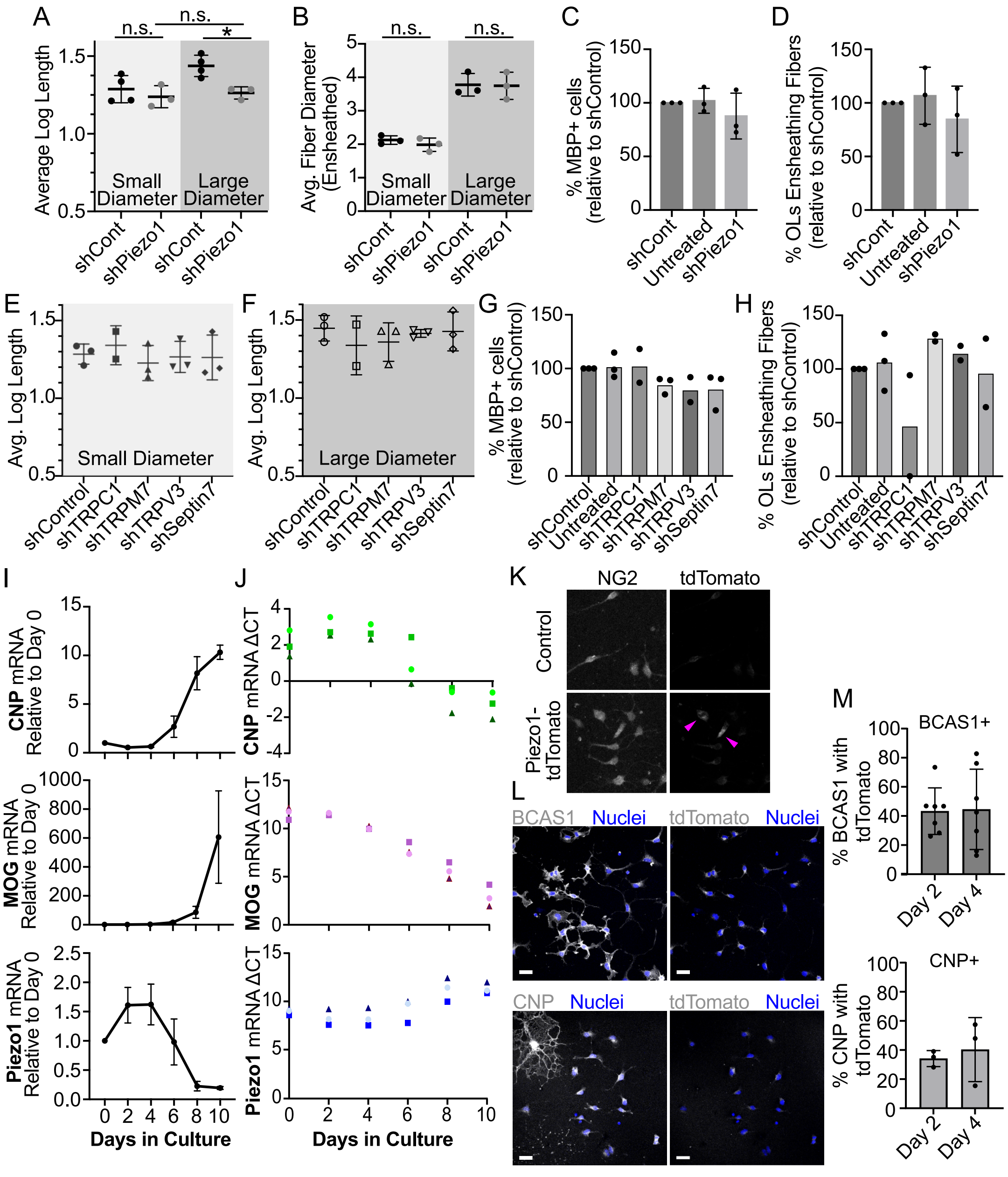

A
n.s.
n.s.
*
Average Log Length
Small Diameter
Large Diameter
shCont
shPiezo1
shCont
shPiezo1
B
n.s.
n.s.
Avg. Fiber Diameter (Ensheathed)
Small Diameter
Large Diameter
C
% MBP+ cells (relative to shControl)
shCont
Untreated
shPiezo1
D
% OLs Ensheathing Fibers (relative to shControl)
E
Avg. Log Length
Small Diameter
shControl
shTRPC1
shTRPM7
shTRPV3
shSeptin7
F
Large Diameter
G
% MBP+ cells (relative to shControl)
Untreated
H
% OLs Ensheathing Fibers (relative to shControl)
I
CNP mRNA Relative to Day 0
MOG mRNA Relative to Day 0
Piezo1 mRNA Relative to Day 0
Days in Culture
J
CNP mRNA ΔCT
MOG mRNA ΔCT
Piezo1 mRNA ΔCT
Days in Culture
K
NG2
tdTomato
Control
Piezo1-tdTomato
L
BCAS1 Nuclei
tdTomato Nuclei
CNP Nuclei
tdTomato Nuclei
M
BCAS1+
% BCAS1 with tdTomato
Day 2
Day 4
CNP+
% CNP with tdTomato
