# Supplementary material for: Myelin sheath lengths in the central nervous system scale to axon diameter via oligodendroglial Piezo1
Source: PLoS Biol. 2026 Sep 21;24(9):e3003992. doi: 10.1371/journal.pbio.3003992 (PMC13592707; doi:10.1371/journal.pbio.3003992)
Supplement: Supplementary file 3 [file pbio.3003992.s003.pdf]

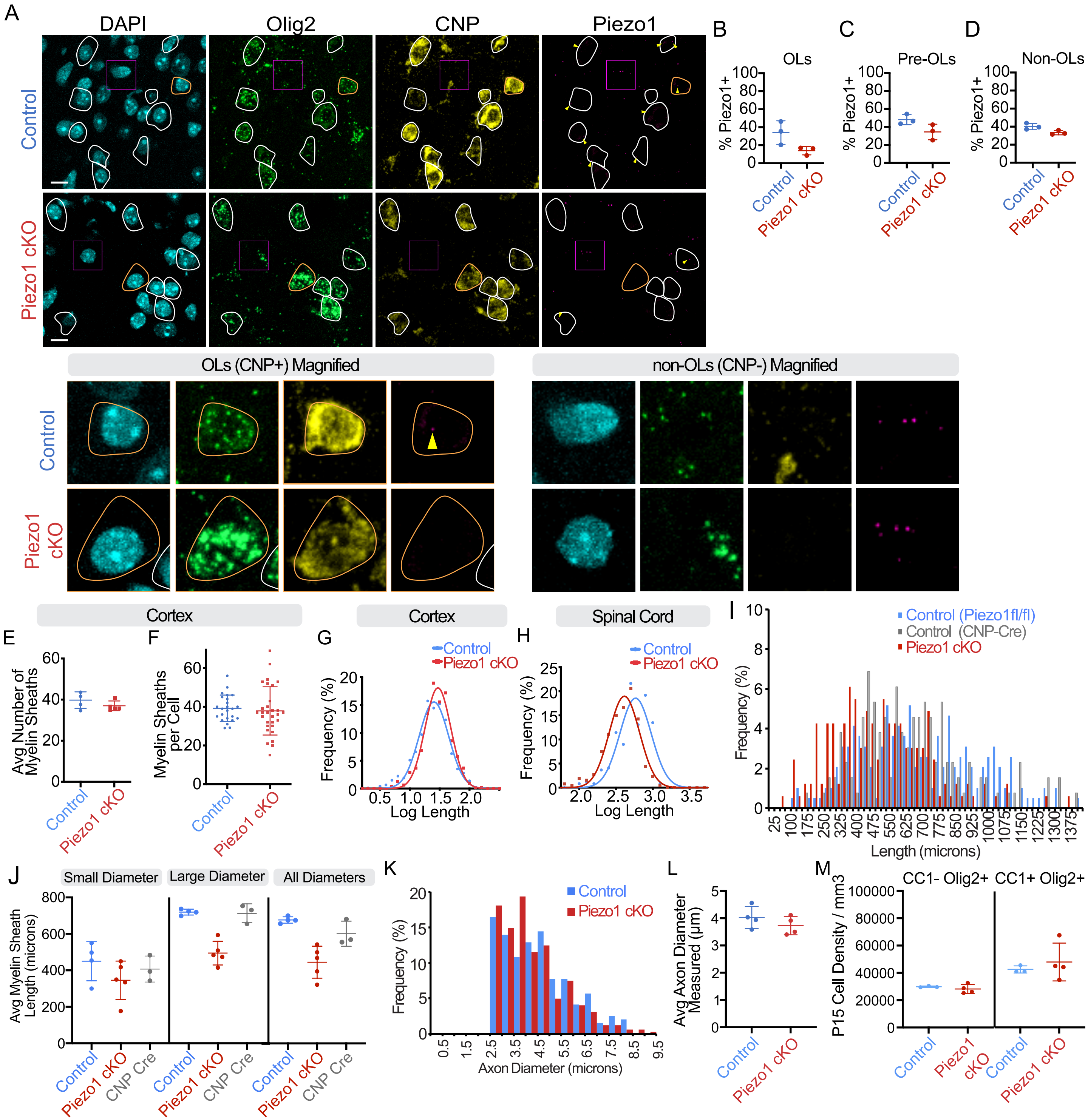

A
DAPI
Olig2
CNP
Piezo1
Control
Piezo1 cKO
B
OLs
C
Pre-OLs
D
Non-OLs
% Piezo1+
OLs (CNP+) Magnified
non-OLs (CNP-) Magnified
Cortex
Cortex
Spinal Cord
E
Avg Number of Myelin Sheaths
F
Myelin Sheaths per Cell
G
Control
Piezo1 cKO
Frequency (%)
Log Length
H
I
Control (Piezo1fl/fl)
Control (CNP-Cre)
Piezo1 cKO
Frequency (%)
Length (microns)
J
Small Diameter
Large Diameter
All Diameters
Avg Myelin Sheath Length (microns)
CNP Cre
K
Frequency (%)
Axon Diameter (microns)
L
Avg Axon Diameter Measured (μm)
M
CC1- Olig2+
CC1+ Olig2+
P15 Cell Density / mm3
