# Supplementary material for: Myelin sheath lengths in the central nervous system scale to axon diameter via oligodendroglial Piezo1
Source: PLoS Biol. 2026 Sep 21;24(9):e3003992. doi: 10.1371/journal.pbio.3003992 (PMC13592707; doi:10.1371/journal.pbio.3003992)
Supplement: Supplementary file 4 [file pbio.3003992.s004.pdf]

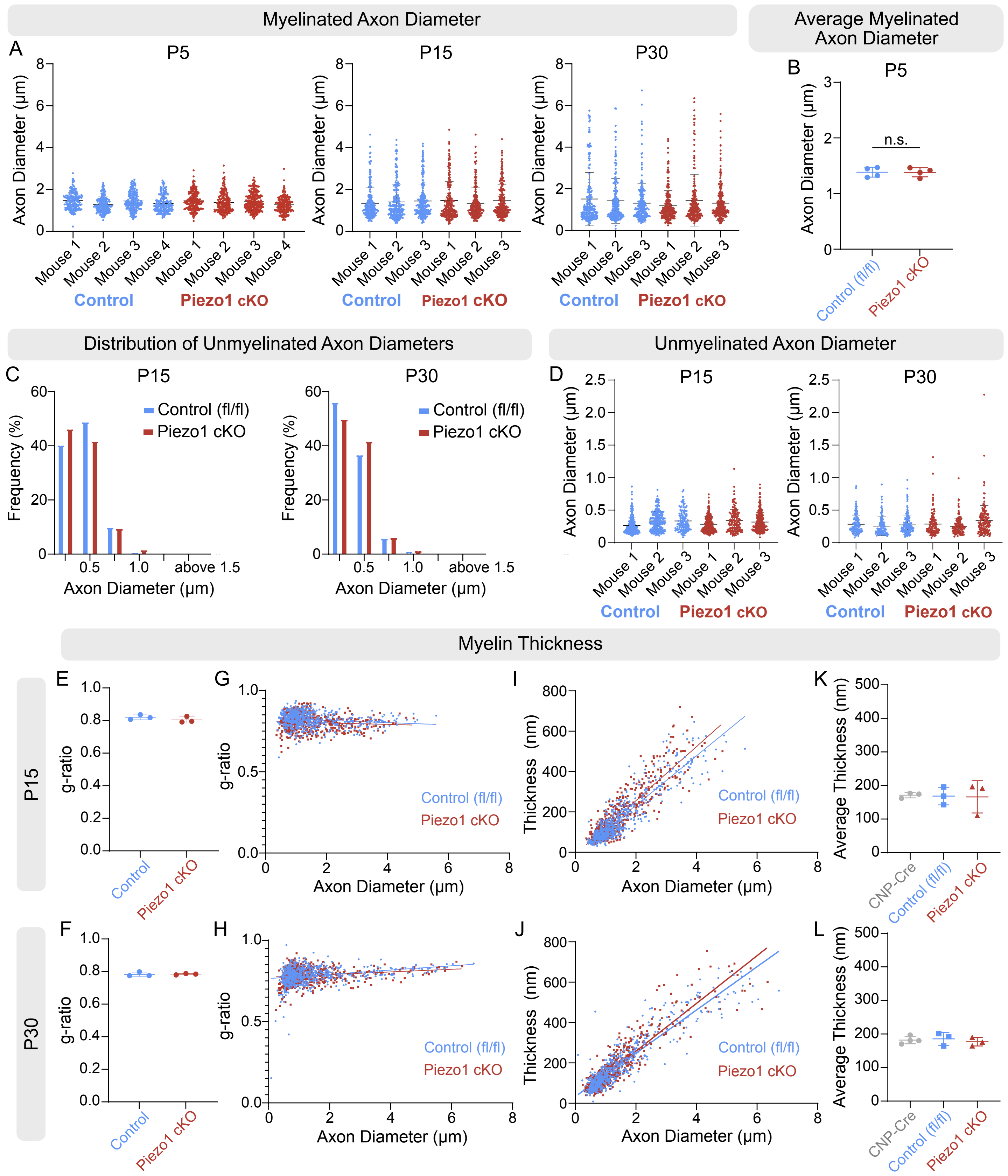
Myelinated Axon Diameter
A
P5
P15
P30
Axon Diameter (µm)
Mouse 1
Mouse 2
Mouse 3
Mouse 4
Control
Piezo1 cKO
Average Myelinated Axon Diameter
B
P5
n.s.
Control (fl/fl)
Piezo1 cKO
Distribution of Unmyelinated Axon Diameters
C
P15
P30
Frequency (%)
Control (fl/fl)
Piezo1 cKO
0.5
1.0
above 1.5
Axon Diameter (µm)
Unmyelinated Axon Diameter
D
P15
P30
Myelin Thickness
P15
P30
E
F
g-ratio
Control
Piezo1 cKO
G
H
I
J
Thickness (nm)
K
L
Average Thickness (nm)
CNP-Cre
Control (fl/fl)
Piezo1 cKO
