# Supplementary material for: Myelin sheath lengths in the central nervous system scale to axon diameter via oligodendroglial Piezo1
Source: PLoS Biol. 2026 Sep 21;24(9):e3003992. doi: 10.1371/journal.pbio.3003992 (PMC13592707; doi:10.1371/journal.pbio.3003992)
Supplement: Supplementary file 5 [file pbio.3003992.s005.docx]

S5 Table. shRNA sequences.

| **TARGET GENE** | **TARGET SEQUENCE** |
| --- | --- |
| TRPC1 | GAATTTAAGTCGTCTGAAA |
|  | CGCAAGCTCGGAATTCTCG |
|  | ACGGTTGTCAGAAACTTAT |
| TRPM7 | AACCGGAGCTTGGATTTTA |
|  | AACAGGGTCCGACTAATCC |
|  | GGAATGATCTTGTTGGGAG |
| TRPV3 | CCCTGACCCTCGTCTCTTA |
|  | CTGTGTCATCCTCACTCTA |
|  | ATTGATAACCGGCATGAGA |
| SEPT7 | CAATAACGTACACTATGAG |
|  | TCCAGAAACGGATGATGAA |
|  | CTGGGAAAGTCGACATTAA |
| PIEZO1 | AAGTACGACCTGGTGCAAC |
|  | TCACGGGCATCTACGTCAA |
|  | TGCTGTGCCTCACGTGTT |
